# A biologically plausible learning rule for the Infomax on recurrent neural networks

**DOI:** 10.3389/fncom.2014.00143

**Published:** 2014-11-25

**Authors:** Takashi Hayakawa, Takeshi Kaneko, Toshio Aoyagi

**Affiliations:** ^1^Department of Morphological Brain Science, Graduate School of Medicine, Kyoto UniversityKyoto, Japan; ^2^CREST, Japan Science and Technology AgencyKawaguchi, Japan; ^3^Department of Applied Analysis and Complex Dynamics, Graduate School of Informatics, Kyoto UniversityKyoto, Japan

**Keywords:** information maximization, biologically plausible learning rule, recurrent neural network, neuronal avalanche, precise firing sequence, orientation selectivity, spike-timing-dependent plasticity

## Abstract

A fundamental issue in neuroscience is to understand how neuronal circuits in the cerebral cortex play their functional roles through their characteristic firing activity. Several characteristics of spontaneous and sensory-evoked cortical activity have been reproduced by Infomax learning of neural networks in computational studies. There are, however, still few models of the underlying learning mechanisms that allow cortical circuits to maximize information and produce the characteristics of spontaneous and sensory-evoked cortical activity. In the present article, we derive a biologically plausible learning rule for the maximization of information retained through time in dynamics of simple recurrent neural networks. Applying the derived learning rule in a numerical simulation, we reproduce the characteristics of spontaneous and sensory-evoked cortical activity: cell-assembly-like repeats of precise firing sequences, neuronal avalanches, spontaneous replays of learned firing sequences and orientation selectivity observed in the primary visual cortex. We further discuss the similarity between the derived learning rule and the spike timing-dependent plasticity of cortical neurons.

## 1. Introduction

The cerebral cortex in mammalian brains plays a central role in higher-order functions such as perception, recognition, planning, and execution of goal-directed behaviors, learning and memory. However, it remains to be understood how these functions are realized through the activity of cortical neurons. Many experiments have been conducted to investigate the underlying computations in cortical circuits, and some of them have revealed the presence of characteristic activity profiles of cortical neurons. In parallel with these experimental observations, computational models have been proposed, reproducing such characteristic activities by simulating the models.

For example, simple and complex cells in the primary visual cortex (V1) of mammalian brains were found to show selective responses to visual stimuli of specific orientations (Hubel and Wiesel, 1959). The response properties of simple cells can be understood as feedforward computations acquired according to some learning principle such as the Infomax principle (Linsker, 1988; Bell and Sejnowski, 1997), the sparse coding principle (Olshausen and Field, 1997; Barlow, 2001) and independent component analysis (ICA) (for a review, see Hyvärinen et al., 2001b). The response properties of complex cells have been reproduced by extending these models of simple cells hierarchically (Hyvärinen et al., 2001a; Karklin and Lewicki, 2005). Based on these guiding learning principles, several authors have constructed biologically plausible learning algorithms in feedforward neural networks that reproduce the response properties of visual neurons including orientation selectivity (Deco and Parra, 1997; Okajima, 2001; Savin et al., 2010; Zylberberg et al., 2011; Tanaka et al., 2012). Hence, Infomax learning has been proposed as one of the candidate learning principles in V1.

Real cortical circuits consist not only of feedforward connections but also of recurrent connections (for a review, see Douglas and Martin, 2007; Kaneko, 2013). In particular, spontaneous activity in the cerebral cortex is thought to be produced by the recurrent connections in the cortical circuits. Repetition of precise firing sequences has been observed in the spontaneous activity of cortical neurons in slice cultures (Ikegaya et al., 2004). Furthermore, task-related sequential firing of cortical neurons in behaving animals and its repetition in the sleeping (Skaggs and McNaughton, 1996; Lee and Wilson, 2002) and quietly awake state (Yao et al., 2007) have been observed *in vivo*. As another one of the characteristic firing profiles of the spontaneous cortical activity, “neuronal avalanche” has also been reported; in certain experimental conditions, the sizes of the bursts in spontaneous bursting activity in the cerebral cortex were shown to obey a power law, and this phenomenon was named “neuronal avalanche” (Beggs and Plenz, 2003).

Historically, the emergence of stereotypical sequential firing activity was predicted in the theory of cell assembly (Hebb, 1949). Guided by this notion, previous studies have shown by simulation that certain network structures of recurrent neural networks allow the emergence of repeats of precise firing sequences and/or neuronal avalanches in the spontaneous activity of the network models (Teramae and Fukai, 2007; Tanaka et al., 2009; Teramae et al., 2012). Teramae et al. (2012) showed that sparsely distributed strong connections in a recurrent neural network lead to apparently asynchronous and irregular spontaneous activity with repeats of precise firing sequences, in accordance with experimental findings (Renart et al., 2010). From the viewpoint of learning, Izhikevich (2006) numerically demonstrated that stereotypical firing sequences appear in a recurrent neural network self-organized by spike-timing-dependent plasticity (STDP), although there is a criticism that his model requires EPSPs of unrealistic sizes to reproduce the firing sequences. Furthermore, Tanaka et al. (2009) recently revealed that the characteristics of spontaneous cortical activity in addition to orientation selectivity in V1 are acquired by self-organization of recurrent neural networks according to Infomax learning principle.

Although Tanaka et al. (2009) suggested Infomax learning as the common learning principle for the characteristics of spontaneous cortical activity and orientation selectivity, they did not show a biologically plausible learning rule for the Infomax learning. If the Infomax learning in Tanaka et al. (2009) is realized in the cortical circuits, there must be a learning rule whose components can possibly be computed through biophysical mechanisms in individual neurons or synapses. However, Tanaka et al. (2009) used a complicated learning algorithm which requires information about firing statistics of the whole network such as inverses of correlation matrices of firing activity. Neurons and synapses cannot have an access to such global information, and thus it is still unclear whether the Infomax learning formulated by Tanaka et al. (2009) in the recurrent settings is biologically plausible. Other types of biological realization of Infomax learning proposed so far (Okajima, 2001; Chechik, 2003; Toyoizumi et al., 2005) do not account for the emergence of spontaneous cortical dynamics. Although several biological mechanisms have been considered for the emergence of the dynamics observed in the cortex (Wörgötter and Porr, 2005; Izhikevich, 2006), to our knowledge, no biologically plausible learning rule in a recurrent neural network has been reported to account for the emergence of both orientation selectivity and the experimentally observed characteristics of spontaneous cortical activity. This might be due to an inherent difficulty in analytically deriving biologically plausible learning rules in recurrent neural networks. However, to understand how the cerebral cortex realizes its highly developed functions, it is the critical first step to discern such a biologically plausible learning rule.

In the present article, we construct a biologically plausible rule for the Infomax learning in a recurrent neural network, and show by numerical simulations that the learning rule reproduces repeats of precise firing sequences, neuronal avalanches, spontaneous replays of learned firing sequences, and orientation selectivity of simple cells. We further discuss the similarity of the derived learning rule to the reward-modulated STDP proposed by Florian (2007), and suggest several candidate neural substrates for its biological realization in cortical circuits.

## 2. Results

### 2.1. A biologically plausible learning rule for the recurrent infomax

In the present study, we consider discrete-time stochastic dynamics of recurrent neural networks similar to those of the preceding study (Tanaka et al., 2009). The recurrent neural network *x*^*t*^ ∈ {0, 1}^*N*^ consists of *N* simple binary neuron models *x*^*t*^_*i*_ with neuron index *i* and time index *t*, each of which takes one of the two states, 1 (fire) or 0 (quiescent). Each neuron receives an input *s*^*t*^_*i*_ from all the other neurons as *s*^*t*^_*i*_ = ∑_*j* ≠ *i*_*w*_*ij*_*x*^*t*^_*j*_ − *h*_*i*_ at time *t*, and it fires at the next time step with a probability of $p\left(x_{i}^{t\;+\;1}=1|x^{t}\right)=p_{\text{max}}\sigma \left(s_{i}^{t}\right)$. Here, we have denoted the logistic function as $\sigma \left(s_{i}^{t}\right)=1/\left\{1+\exp\left(-s_{i}^{t}\right)\right\}$, and parametrized the maximal probability of firing transmission with *p*_max_. The model parameters {*w*_*ij*_}_1 ≤ *j* ≤ *N*_ and *h*_*i*_ represent the synaptic weights and the firing threshold of the postsynaptic neuron *i*, respectively, and are updated by the learning rule derived below. These settings may be understood as a discrete approximation of continuous-time neuronal dynamics (see Methods). Following the model settings in the previous studies of biologically plausible learning rules in neural networks (Savin et al., 2010; Zylberberg et al., 2011; Frémaux et al., 2013), we allow each neuron to make both of excitatory and inhibitory synapses. For the precise modeling of interactions between excitatory and inhibitory neurons, we must consider various experimental findings, such as the existence of many different kinds of inhibitory neurons (Markram et al., 2004) and excitatory axo-axonic synapses on axons of inhibitory neurons (Ren et al., 2007). Although such precise modeling is apparently intractable and beyond the scope of the current study, we can consider possible biological realization of our idealized neurons with excitatory and inhibitory synapses, for example, by replacing a single neuron with a pair of excitatory and inhibitory neurons which have the same responsiveness (see Discussion for the details). Thus, the usage of such idealized neurons does not critically affect the biological plausibility.

Originally, Infomax learning was defined as the maximization of mutual information between two groups of neural elements (Linsker, 1988; Bell and Sejnowski, 1997), and many extensions have been considered (Kay and Phillips, 2010). The mutual information was defined according to information theory (Cover and Thomas, 2012). In Tanaka et al. (2009), assuming the convergence of distributions of firing activity *x*^*t*^ to stationary distributions *p*_*s*_(*x*^*t*^), the authors proposed to define recurrent Infomax as the maximization of mutual information between the firing activities at two successive time steps, under the constraint that the mean firing rates of neurons must be a fixed small value *p*_0_ ≪ 1:

(1) $$\begin{array}{l} \underset{w_{ij},h_{i}}{\max}\text{I}\left[x^{t};x^{t-1}\right]=\sum_{x^{t},x^{t-1}}p_{s}\left(x^{t},x^{t-1}\right)\log\frac{p\left(x^{t}|x^{t-1}\right)}{p_{s}\left(x^{t}\right)},\\ \;sub.\;to\text{ E}\left[x_{i}^{t}\right]=p_{0}\ll 1. \end{array}$$

Since the brain needs to retain information about its past activity, the above definition of the recurrent Infomax learning is straightforward.

Tanaka and colleagues did not discuss, however, plausible mechanisms for the recurrent Infomax learning in the brain. Thus, we derive a biologically plausible learning rule, which predicts possible mechanisms for the recurrent Infomax learning in real cortical circuits. Since it is thought to be impossible for a neural network to directly compute the mutual information itself, some approximation is needed. Thus, we are going to derive the learning rule from the following approximate objective function:

(2) $$\begin{array}{l} A=\underset{A_{1}}{\underset{︸}{\sum_{i}\log\text{I}\left[x_{i}^{t};x^{t-1}\right]}}-\underset{A_{2}}{\underset{︸}{\kappa \sum_{i<j}\left({\langle x_{i}^{t}x_{j}^{t}\rangle }_{\infty }-{\langle x_{i}^{t}\rangle }_{\infty }{\langle x_{j}^{t}\rangle }_{\infty }\right)}}\\ \;-\underset{A_{3}}{\underset{︸}{\frac{\eta }{2}\sum_{i}{\left({\langle x_{i}^{t}\rangle }_{\infty }-p_{0}\right)}^{2}}}-\underset{A_{4}}{\underset{︸}{\frac{\zeta }{2}\sum_{i}{\langle {\left(s_{i}^{t}-s_{0}\right)}^{2}\rangle }_{\infty }}}. \end{array}$$

Here, the angle brackets with subscript ∞ represent the long-time averages of their arguments. Throughout this paper, assuming ergodicity, we identify long-time averages with corresponding ensemble averages as

$${\langle x^{t}\rangle }_{\infty }=\underset{T\to \infty }{\lim}\frac{1}{T}\sum_{t=1}^{T}x^{t}=\sum_{x^{t}}x^{t}p_{s}\left(x^{t}\right).$$

The role of each term in Equation (2) is as follows (see Methods for further details of the construction of the objective function *A*). Predictability term *A*_1_ = ∑_*i*_ log I [*x*^*t*^_*i*_; *x*^*t* − 1^] represents how predictable the firing activity *x*^*t*^ is from the firing activity at previous time steps *x*^*t* − 1^. In other words, this term forces neurons to fire deterministically as far as possible, that is, to fire with high probabilities near *p*_max_ in response to specific inputs, and not to fire at all for the other inputs. This term mathematically provides a lower bound of the mutual information I [*x*^*t*^; *x*^*t* − 1^] if and only if the firing of each neuron *x*^*t*^_*i*_ (1 ≤ *i* ≤ *N*) is independent. We, therefore, impose a penalty based on the sum of the pairwise correlations, correlation term $A_{2}=\kappa \sum_{i\;<\;j}\left({\langle x_{i}^{t}x_{j}^{t}\rangle }_{\infty }-{\langle x_{i\text{​}}^{t}\rangle }_{\infty }{\langle x_{j}^{t}\rangle }_{\infty }\right)$, so as to bound the firing activity near the independent distribution. The correlation term *A*_2_ is also interpreted as a penalty term for population sparseness, noting that $\sum_{i\;<\;j}x_{i}^{t}x_{j}^{t}=\frac{1}{2}m^{t}\left(m^{t}-1\right)$ where *m*^*t*^ = ∑_*i*_*x*^*t*^_*i*_. Firing-rate term $A_{3}=\frac{\eta }{2}\sum_{i}{\left({\langle x_{i}^{t}\rangle }_{\infty }-p_{0}\right)}^{2}$ is a penalty term for controlling the average firing rates of all the neurons in the network to be *p*_0_. Since *s*^*t*^_*i*_ can be interpreted as an input current to the neuron *i* at each time step (see Methods and **Figure 8**), its fluctuation should be confined within a physiologically reasonable range. We therefore impose an additional penalty for excessively large fluctuation of *s*^*t*^_*i*_ with fluctuation term $A_{4}=\frac{\zeta }{2}\sum_{i}{\langle {\left(s_{i}^{t}-s_{0}\right)}^{2}\rangle }_{\infty }$. The reference input strength $s_{0}=\log\left(\frac{p_{0}}{p_{\text{max}}-p_{0}}\right)$ is determined so that neurons fire with a probability of $p\left(x_{i}^{t+1}=1|s_{i}^{t}=s_{0}\right)=p_{0}$ when the strength of the inputs to the neurons is *s*_0_. If excessively large fluctuation of the inputs is allowed, a kind of singularity appears in the dynamics and the approximation with the pairwise correlations fails (see Methods and **Figure 7**).

Then, we construct a biologically plausible learning rule as a stochastic gradient ascent algorithm for the objective function, Equation (2):

(3) $$\begin{array}{l} w_{ij}^{t}\leftarrow w_{ij}^{t-1}+\epsilon \frac{\tau }{T}\left(\gamma_{1}^{t}-\gamma_{2}^{t}-\gamma_{3}^{t}-\gamma_{4}^{t}\right){\langle \psi_{i}^{t}x_{j}^{t}\rangle }_{\tau }\\ \;-\epsilon \frac{\zeta }{T}\left(s_{i}^{t}-s_{0}\right)x_{j}^{t}, \end{array}$$

(4) $$\begin{array}{l} h_{i}^{t}\leftarrow h_{i}^{t-1}-\epsilon \frac{\tau }{T}\left(\gamma_{1}^{t}-\gamma_{2}^{t}-\gamma_{3}^{t}-\gamma_{4}^{t}\right){\langle \psi_{i}^{t}\rangle }_{\tau }\\ \;+\epsilon \frac{\zeta }{T}\left(s_{i}^{t}-s_{0}\right), \end{array}$$

where

$$\begin{array}{l} \gamma_{1}^{t}=\sum_{i}\frac{1}{{\langle \log\frac{p(x_{i}^{t}|x^{t-1})}{Z_{i}^{t}}\rangle }_{T}}\log\frac{p\left(x_{i}^{t}|x^{t-1}\right)}{Z_{i}^{t}},\\ \gamma_{2}^{t}=\kappa \{\frac{1}{2}m^{t}\left(m^{t}-1\right)-\left({\langle m^{t}\rangle }_{T}-p_{0}\right)m^{t}\},\\ \gamma_{3}^{t}=\eta \sum_{i}\left({\langle p_{\text{max}}\sigma \left(s_{i}^{t}\right)\rangle }_{T}-p_{0}\right)x_{i}^{t},\\ \gamma_{4}^{t}=\frac{\zeta }{2}\sum_{i}{\left(s_{i}^{t}-s_{0}\right)}^{2},\\ Z_{i}^{t}=\{\begin{array}{c} \begin{array}{l} \;{\langle p_{\text{max}}\sigma \left(s_{i}^{t}\right)\rangle }_{T},\text{ if }x_{i}^{t}=1,\\ 1-{\langle p_{\text{max}}\sigma \left(s_{i}^{t}\right)\rangle }_{T},\text{ if }x_{i}^{t}=0, \end{array} \end{array}\\ \begin{array}{c} \psi_{i}^{t}=\left\{ \begin{array}{l} \;1-\sigma \left(s_{i}^{t}\right)\;,\text{ if }x_{i}^{t+1}=1,\\ -\frac{p_{\text{max}}\sigma \left(s_{i}^{t}\right)\left(1-\sigma \left(s_{i}^{t}\right)\right)}{1-p_{\text{max}}\sigma \left(s_{i}^{t}\right)},\text{ if }x_{i}^{t+1}=0, \end{array}\right. \end{array}\\ m^{t}=\sum_{i}x_{i}^{t}\;. \end{array}$$

We remove the singularity at 〈 log {*p*(*x*^*t*^_*i*_|*x*^*t* − 1^)/*Z*^*t*^_*i*_} 〉_*T*_ = 0 by replacing it with δ = 1.0× 10^−3^ if 〈 log {*p*(*x*^*t*^_*i*_|*x*^*t* − 1^)/*Z*^*t*^_*i*_} 〉_*T*_ ≤ δ. The angle brackets with subscript τ and *T* are calculated recursively at each time step as, for example,

$${\langle m^{t}\rangle }_{T}=\frac{1}{T}m^{t-1}+\left(1-\frac{1}{T}\right){\langle m^{t-1}\rangle }_{T}.$$

Then, ${\langle m^{t}\rangle }_{T}=\sum_{u\;=\;0}^{\infty }\frac{1}{T}{\left(1-\frac{1}{T}\right)}^{u}m^{t\;-\;u\;-\;1}$ is interpreted as a leaky integration of the past amounts *m*^*t* − *u* − 1^, (*u* ≥ 0) with a leak constant *T*. As *T* → ∞ and the process under consideration is stationary, 〈 *m*^*t*^〉_*T*_ approaches the stationary average of *m*^*t*^.

**Figure 1:**
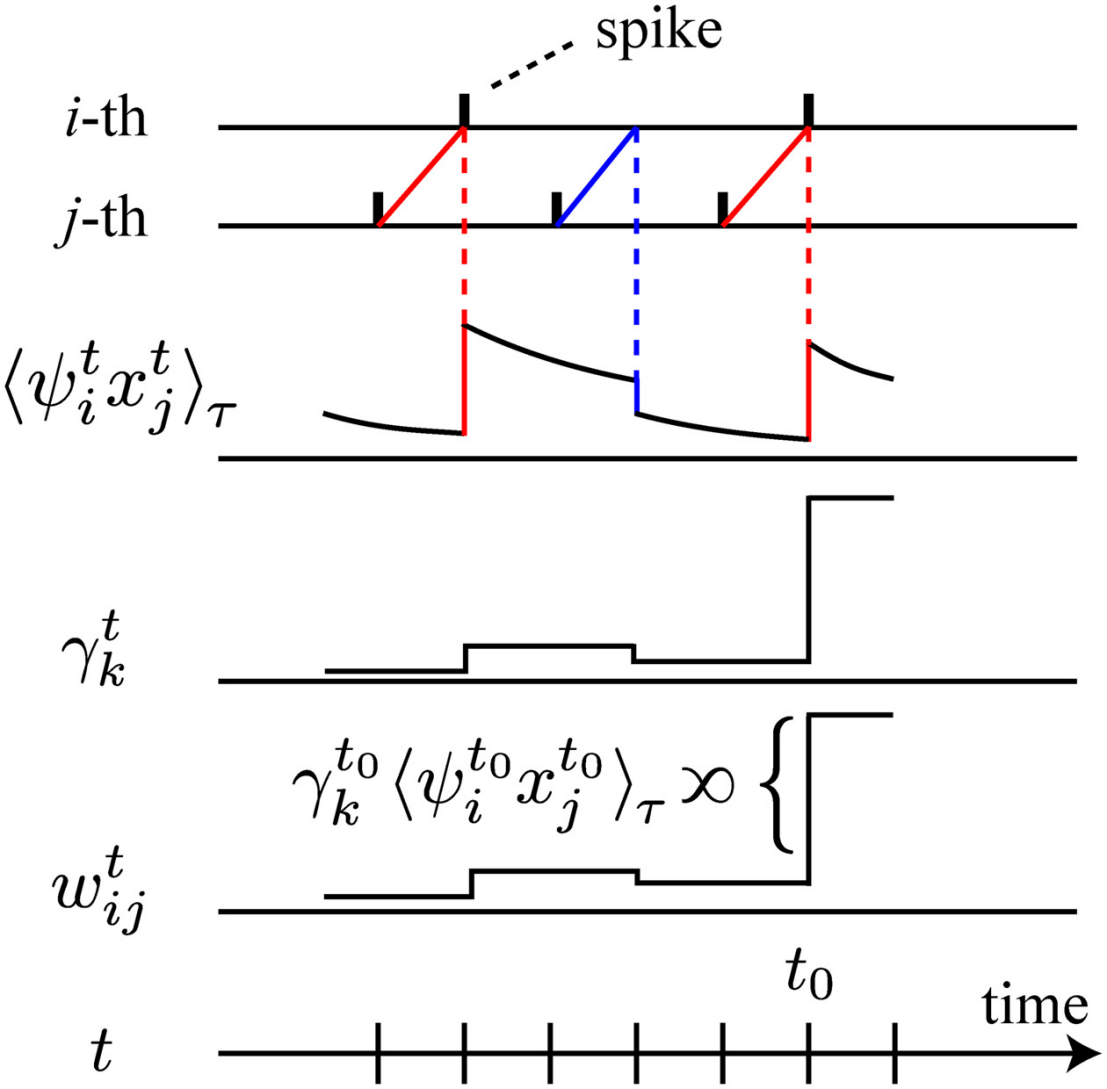
**Schematic illustration of the learning rule**. For simplicity, we illustrate only the relation between ${\langle \psi_{i}^{t}x_{j}^{t}\rangle }_{\tau }$, γ^*t*^_*k*_ and *w*^*t*^_*ij*_, omitting the other components of the learning rule. On the first two lines, spikes of the *i*-th and the *j*-th neurons are depicted. On the third line, ${\langle \psi_{i}^{t}x_{j}^{t}\rangle }_{\tau }$ decays with time constant τ, and is modulated by the interactions between the spiking activity of the *i*-th and the *j*-th neurons. When the firing of the presynaptic *j*-th neuron precedes the firing of the postsynaptic *i*-th neuron, ${\langle \psi_{i}^{t}x_{j}^{t}\rangle }_{\tau }$ increases (red line). If the postsynaptic neuron fails to fire, ${\langle \psi_{i}^{t}x_{j}^{t}\rangle }_{\tau }$ decreases (blue line). The change of the synaptic weight *w*_*ij*_ at time *t*_0_ is proportional to the product of the amount of global signal γ^*t*_0_^_*k*_ and ${\langle \psi_{i}^{t_{0}}x_{j}^{t_{0}}\rangle }_{\tau }$ as depicted on the fifth line.

In the above learning rule, we notice that all terms except γ^*t*^_*k*_ on the right-hand sides of Equations (3) and (4) can be computed by the postsynaptic neuron *i* based on its own activity and local interactions with the other neurons. Thus, these terms are biologically plausible. It should be particularly noted that the temporal integration such as ${\langle \psi_{i}^{t}x_{j}^{t}\rangle }_{\tau }$ can be realized locally at each synapse or each neuron, possibly with very large leak constants τ and *T* (phosphorylation or gene expression may be considered). In the limit of τ → ∞ and *T*/τ → ∞, the stochastic approximation of the gradient ascent is exact. In addition to these local processes, it suffices for the biological plausibility of the overall learning rule to assume the existence of neural substrates corresponding to the global signals γ^*t*^_*k*_ (1 ≤ *k* ≤ 4). We are able to consider a scenario for such neural substrates as follows. For γ^*t*^_1_, γ^*t*^_3_, and γ^*t*^_4_, we consider a rapidly diffusing substrate emitted by each neuron (nitric oxide, neuropeptides, lipid metabolites, endcannabinoids, and so on), the sum of whose amount is γ^*t*^_1_ − γ^*t*^_3_ − γ^*t*^_4_, since γ^*t*^_*k*_ (*k* = 1, 3, 4) are sums of locally computable quantities over the neuronal population. For γ^*t*^_2_, we need to consider another substrate, since it is a non-linear function of population activity *m*^*t*^ and its temporal integration 〈 *m*^*t*^〉_*T*_. As a candidate neural substrate, we can consider interneurons that are able to monitor the overall activity *m*^*t*^ of the network and to return a non-linear feedback to pyramidal neurons. As shown in Figure 1, the action of these two types of neural substrates is to amplify the leaky integration of the past local quantities ψ^*t* − *u*^_*i*_*x*^*t* − *u*^_*j*_ and ψ^*t* − *u*^_*i*_ (*u* ≥ 1) by their magnitudes γ^*t*^_*k*_. Each neuron *i* realizes this amplification through its intracellular signaling pathway, receiving the neural substrates corresponding to γ^*t*^_*k*_. We will discuss further detailed scenarios and their plausibility in the Discussion section. In addition, the derivation of this type of a stochastic gradient ascent method is mathematically the same as that for reward-modulated STDP, a neural implementation of reinforcement learning (Florian, 2007; Frémaux et al., 2013) (see Discussion).

In the following sections, we will show the results of numerical simulations of the above learning rule, in which we start from a network with weak random connections taken from a uniform distribution *w*_*ij*_ ~ [−0.1, 0.1] representing synapses in the early developmental stage just after synaptic formation is made, and with *h*_*i*_ = log {(*p*_*max*_ − *p*_0_)/*p*_0_}. With these initial values, the neurons fire almost independently with probability *p*_0_. Then, we update *w*_*ij*_ and *h*_*i*_ according to the learning rule, Equations (3) and (4). We make sure that the results in the following sections are robustly reproduced, with different series of random numbers used in the determination of initial model parameters and in the simulation. We show the learning parameters used for each simulation at the end of the corresponding figure legend. We show the learning parameters in the objective function Equation (2) with the scaled parameters *c*_κ_, *c*_η_, and *c*_ζ_ so that we can see their magnitudes independently of the system size and the average firing rate (see Methods for further details).

(5) $$\kappa =\frac{2}{(N-1)c_{\kappa }p_{0}^{2}},\;\eta =\frac{1}{c_{\eta }^{2}p_{0}^{4}},\;\zeta =\frac{1}{c_{\zeta }^{2}}.$$

To check whether the mutual information is actually maximized, we calculate an approximate measure of the mutual information,

(6) $${\hat{I}}_{gauss}=\log_{2}|\hat{C}|-\frac{1}{2}\log_{2}|\hat{D}|.$$

Here, $\hat{C}$ and $\hat{D}$ are empirical covariance matrices of *x*^*t*^ and *x*^*t*^ ⊗ *x*^*t* − 1^, respectively (see Methods). This estimate $\hat{I}$_*gauss*_ approximates the mutual information by regarding the firing distribution *p*_*s*_(*x*^*t*^) and *p*_*s*_(*x*^*t*^, *x*^*t* − 1^) as Gaussian distributions. It was shown in the numerical experiment in the previous study (Tanaka et al., 2009) that these values provide a good approximation of the exact values of the mutual information.

### 2.2. Reproduction of repeated firing sequences and neuronal avalanches

In this section, we reproduce repeats of precise firing sequences and neuronal avalanches similar to those observed in experimental studies (Beggs and Plenz, 2003; Ikegaya et al., 2004), which have been suggested as a consequence of the maximization of information retention in previous studies (Tanaka et al., 2009; Chen et al., 2010).

We apply the learning rule to a spontaneously firing recurrent neural network *x*^*t*^ consisting of fifty neurons that does not receive external inputs. Then, we observe two typical behaviors of the model after learning, depending on the model parameters: maximal transmission probability *p*_*max*_ and average firing rate *p*_0_.

After learning with larger values of *p*_*max*_ and *p*_0_, we observe a variety of repeated firing sequences in apparently asynchronous and irregular firing activity of the model. According to the theory in the previous study (Tanaka et al., 2009), the relationship between the repeated precise sequences and the mutual information is understood as follows. Following the definition and the notation of entropy and conditional entropy in information theory (Cover and Thomas, 2012), the mutual information can be decomposed as

(7) $$\text{I}\left[x^{t};x^{t-1}\right]=\text{H}\left[x^{t}\right]-\text{H}\left[x^{t}|x^{t-1}\right].$$

**Figure 2:**
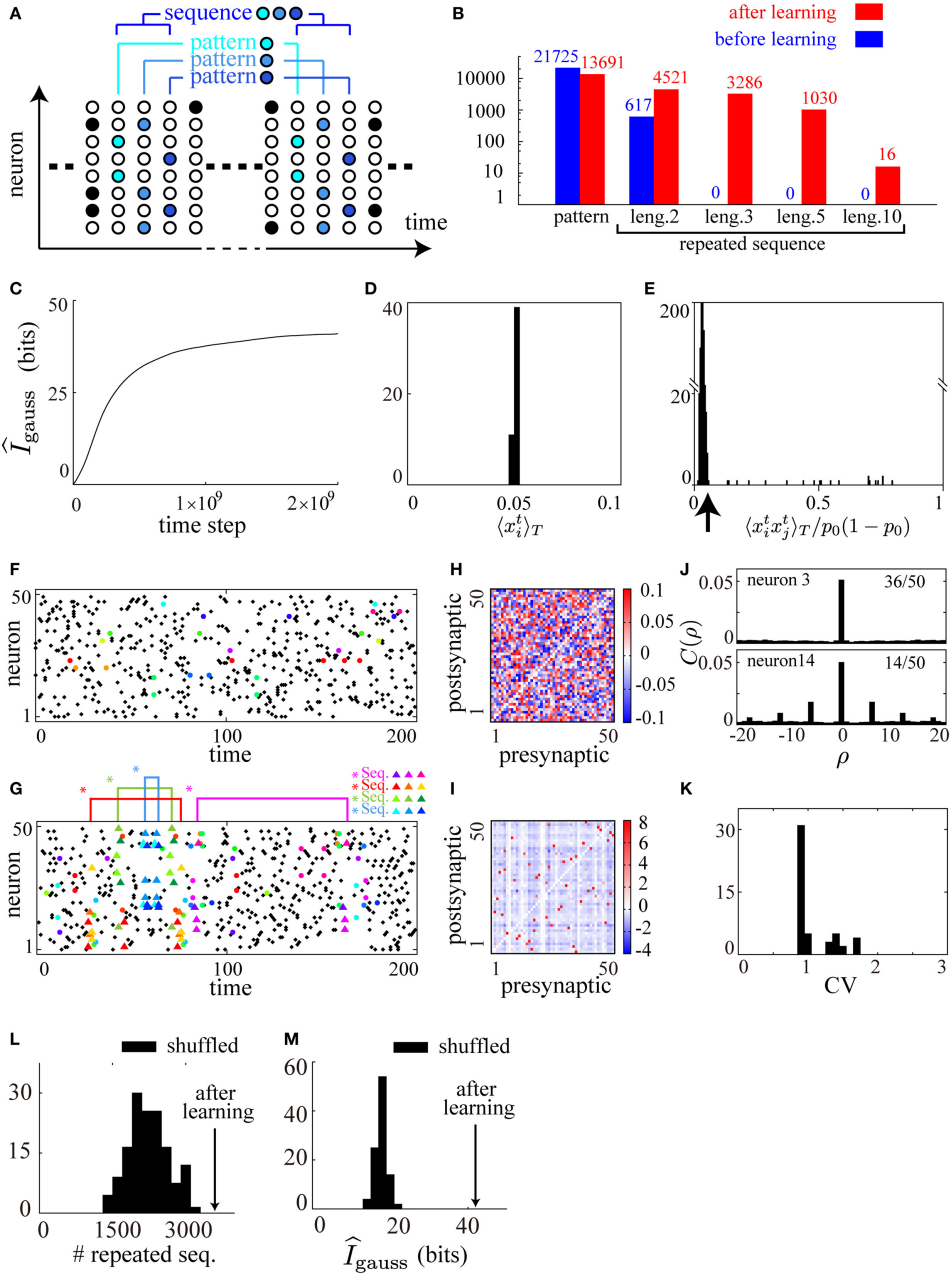
**Cell-assembly-like repeated sequences of firing patterns are reproduced by applying the learning rule to a spontaneously firing recurrent neural network**. **(A)** A schematic illustration of the definition of firing patterns and of sequences of firing patterns. Each color represents a unique firing pattern *x*^*t*^ ∈ {0, 1}^*N*^. A repeated sequence of firing patterns of length 3 {*x*^*t*^, *x*^*t* − 1^, *x*^*t* − 2^} ∈ {0, 1}^3*N*^ is also illustrated. **(B)** Comparison of the numbers of firing patterns and repeated sequences (repeated more than twice) of length 2, 3, 5, and 10 before and after learning, counted for a particular 50000 time steps in the simulation. **(C)** The approximate measure of the mutual information $\hat{I}$_*gauss*_ is calculated during the course of learning. **(D)** A histogram of mean firing rates of the neurons in the network after learning. **(E)** A histogram of pairwise correlations between all the pairs of neurons in the network after learning. **(F,G)** Typical raster plots before **(F)** and after **(G)** learning in which repeated sequences are represented as triangles of different colors, repeated patterns as filled circles of different colors, and other spikes as black dots, according to the definition illustrated in **(A)**. **(H,I)** Connection weight matrices of the network before **(H)** and after **(I)** learning in which rows correspond to connections to single postsynaptic neurons while columns correspond to connections from single presynaptic neurons. **(J)** Typical two examples from the autocorrelograms calculated from the activity of each neuron in the network after learning. Thirty-six of the neurons show no autocorrelation as seen in the upper figure, while fourteen show damped periodicity as seen in the lower figure. **(K)** A histogram of CV values calculated from the activity of each neuron in the network after learning. **(L,M)** Comparison of $\hat{I}$_*gauss*_ **(L)** and the numbers of sequences of length 3 (counted for a particular 50000 time steps) **(M)** of the network after learning (arrows) with those of one hundred shuffled networks (histograms). The following parameters have been used in this simulation: ϵ = 0.006, *c*_η_ = 1.5, *c*_κ_ = 1.0, *c*_ζ_ = 3.0, *p*_0_ = 0.05, *p*_*max*_ = 0.95, *N* = 50, τ = 15, *T* = 50000.

In the above equation, the first term of the right-hand side represents the abundance of firing patterns and the second term represents the predictability of firing activity *x*^*t*^ from *x*^*t* − 1^. Thus, repeats of many long sequences of firing patterns are expected to emerge after the maximization of the mutual information. Here, we have defined a firing pattern as a specific configuration of firing activity *x*^*t*^ ∈ {0, 1}^*N*^, and a sequence of firing patterns of length *L* as a specific configuration of a series of firing patterns {*x*^*t*^, *x*^*t* − 1^, ·s *x*^*t* − *L* + 1^} ∈ {0, 1}^*NL*^ (illustrated in Figure 2A).

In the numerical simulation, by counting the number of firing patterns and sequences during a particular 50000 steps, we find that the numbers of repeated sequences of length 3, 5, and 10 after learning are far larger than those before learning, while the variety of firing patterns after learning is kept high (Figure 2B). During the course of learning, we have checked that the estimate of the mutual information, Equation (6), monotonically increases and tends to saturate (Figure 2C). In addition, we have checked after learning that the average firing rate of each neuron is controlled to be *p*_0_, and that most of the pairwise correlations are controlled to be small, as shown in the histograms in Figures 2D,E. We show typical raster plots of 250 time steps before (Figure 2F) and after (Figure 2G) learning, along with matrices of the synaptic weights before (Figure 2H) and after (Figure 2I) learning. No sequence of length 3 repeats within Figure 2F, while four different repeated sequences of length 3 represented by colored triangles are found within Figure 2G, indicating the abundance of repeated sequences after learning. In contrast, the abundance of repeated patterns represented by colored circles are comparable between Figures 2F,G. These results are consistent with the theoretical consideration based on Equation (7) that a rich variety of repeated sequences should occur after the maximization of the mutual information, suggesting that our model has learned successfully.

In spite of the abundance of repeated firing sequences, the firing activity after learning is apparently asynchronous and irregular (Figure 2G) as observed in the real cortical activity (Renart et al., 2010). Examining the underlying connectivity, we find that the initial small random connections shown in Figure 2H grow into the sparsely distributed strong excitatory and inhibitory connections shown in Figure 2I. In order to quantify the asynchrony and the irregularity, we calculate autocorrelograms Figure 2J and coefficients of variation (CV) of inter-spike intervals (ISIs) Figure 2K for each neuron after learning. In the autocorrelograms, we find almost no autocorrelation for the majority (36/50) of the neurons (the upper graph in Figure 2J), and rapidly damped periodicity for the other neurons (the lower graph in Figure 2J). We find, however, that all of the CV values (Figure 2K) are greater than 1.0 (the value for a Poissonian spike train) indicating that there is no global periodicity of the network and the overall firing activity is irregular. In parallel with our results, a preceding study demonstrated that randomly connected neural networks with sparsely distributed strong synapses display asynchronous and irregular firing activity in which many precise firing sequences are embedded (Teramae et al., 2012). To compare the network after learning with networks with random connections of sparsely distributed strong synapses, we examine the activities of the network after learning and one hundred networks whose synaptic weights are shuffled sets of the original network after learning. Then, the number of repeated sequences in the original network is largest among the shuffled networks (Figure 2L) at the same time as its estimated mutual information is maximal (Figure 2M). Although we are not able to further characterize the statistical properties of the connectivity after learning because of limitations on the system size, the above result suggests that the network structure after learning is more finely tuned for efficient information transmission based on precise firing sequences than random networks with sparsely distributed strong connections.

**Figure 3:**
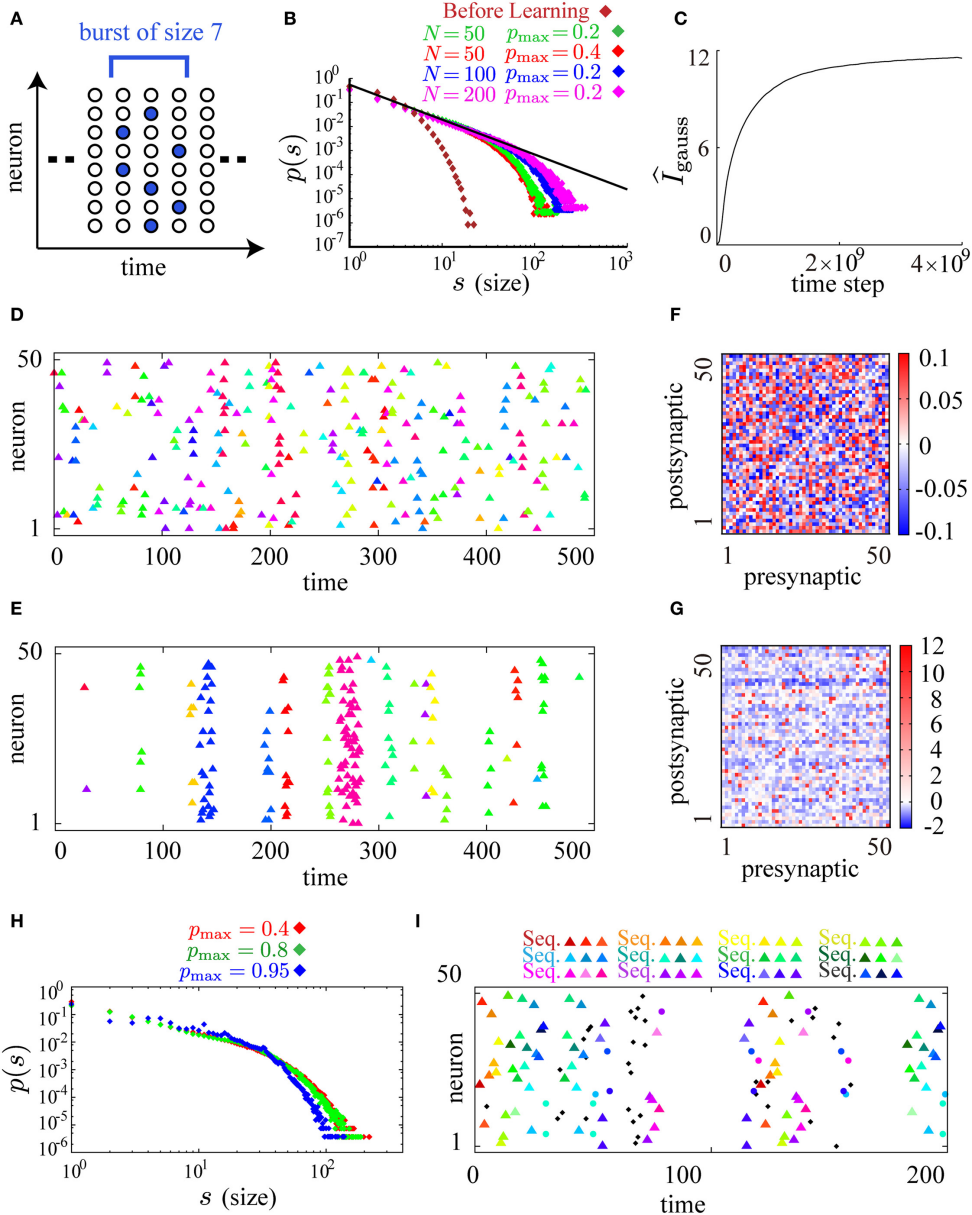
**Neuronal avalanches are reproduced by applying the learning rule to spontaneously firing recurrent neural networks**. **(A)** A schematic illustration of the definition of bursts and their sizes. A burst of size 7 that lasts for 3 time steps is illustrated in the figure. **(B)** Log-log plots of burst size *s* against the occurrence of bursts of that size *p*(*s*) before (brown) and after learning (red for *p*_max_ = 0.4 and *N* = 50, green for *p*_max_ = 0.2 and *N* = 50, blue for *p*_max_ = 0.2 and *N* = 100, and magenta for *p*_max_ = 0.2 and *N* = 200). The size distributions after learning fall on the straight line of slope around −3/2 (the black line). **(C)** The approximate measure of the mutual information $\hat{I}$_*gauss*_ is calculated during the course of learning. **(D,E)** Typical raster plots before **(D)** and after **(E)** learning in which spikes belonging to different bursts are displayed in different colors. **(F,G)** Connection weight matrices of the network before **(F)** and after **(G)** learning in which rows correspond to connections to single postsynaptic neurons while columns correspond to connections from single presynaptic neurons. **(H)** Log-log plots of burst size *s* against the occurrence of bursts of that size *p*(*s*) for higher *p*_max_ ( = 0.8, 0.95) along with the plot for *p*_max_ = 0.4. **(I)** A typical raster plot after learning with *p*_max_ = 0.95. Colored triangles and circles indicates repeated firing sequences of length 3 and repeated firing patterns, respectively, as in Figure 2G. Repetition of as many as twelve firing sequences can be seen in the figure. The following sets of learning parameters have been used in this simulation: ϵ = 0.02, *c*_η_ = 10.0, *c*_κ_ = 30.0, *c*_ζ_ = 3.0, *p*_0_ = 0.01, *p*_max_ = 0.2, 0.4, 0.8, *N* = 50, τ = 10, *T* = 50000; ϵ = 0.01, *c*_η_ = 20.0, *c*_κ_ = 60.0, *c*_ζ_ = 4.0, *p*_0_ = 0.005, *p*_max_ = 0.2, *N* = 100, τ = 15, *T* = 50000; ϵ = 0.006, *c*_η_ = 40.0, *c*_κ_ = 100.0, *c*_ζ_ = 6.0, *p*_0_ = 0.0025, *p*_max_ = 0.2, *N* = 200, τ = 20, *T* = 50000; ϵ = 0.005, *c*_η_ = 3.0, *c*_κ_ = 1.0, *c*_ζ_ = 10.0, *p*_0_ = 0.01, *p*_max_ = 0.95, *N* = 50, τ = 20, *T* = 50000.

After learning with smaller values of *p*_*max*_ and *p*_0_, the firing activity of the model is similar to neuronal avalanche. Following the previous theoretical and experimental studies of neuronal avalanche (Beggs and Plenz, 2003; Tanaka et al., 2009), we define a single burst as a cluster of firing partitioned by empty time steps without firing and its size as the number of spikes in that cluster (illustrated in Figure 3A). By plotting burst size *s* against the occurrence *p*(*s*) of bursts of that size on the log-log scale, we find that *p*(*s*) obeys a power law with a universal exponent around −3/2 for different parameters *p*_*max*_ = 0.4, 0.2 and different system sizes *N* = 50, 100, 200 after learning while *p*(*s*) before learning decreases rapidly as *s* increases (Figure 3B). During the process of learning, we have checked that the estimate of the mutual information, Equation (6), monotonically increases (Figure 3C), suggesting that the mutual information is successfully maximized. We show typical raster plots before (Figure 3D) and after (Figure 3E) learning. Representing different bursts in different colors, we observe that the initial random firing activity (Figure 3D) has become apparently bursty activity after learning (Figure 3E). Examining the underlying connectivity, we find that the initial small random connections in Figure 3F grow into the sparsely distributed strong excitatory and inhibitory connections shown in Figure 3G. In Figure 3G, most neurons feed two or three strong excitatory inputs to other neurons, probably reflecting the following requirement. $\text{E}\left[\sum_{i}x_{i}^{t}|\sum_{i}x_{i}^{t\;-\;1}=n\right]=n$ is necessary for the emergence of neuronal avalanches, when the firing activity {*x*^*t*^_*i*_}_1 ≤ *i* ≤ *N*_ is controlled to be almost independent by the correlation term *A*_2_ (Zapperi et al., 1995). Then, a single firing in the network needs to evoke a single firing on average at the next time step. Given that *p*_*max*_ = 0.4, and that $p\left(x_{i}^{t+1}=1|x^{t}\right)$ must be *p*_*max*_ or 0 for the maximization of the predictability term *A*_1_, each neuron needs to feed strong connections to 1/0.4 = 2.5 target neurons on average. Although these results are robustly observed for a wide range of model parameters, the power law is slightly distorted for higher maximal firing probabilities (the green plot for *p*_*max*_ = 0.8 in Figure 3H). When *p*_*max*_ is set to be near 1.0, the size distribution *p*(*s*) seems to have small multiple peaks (the blue plot for *p*_*max*_ = 0.95 in Figure 3H). A typical raster plot of 200 time steps after learning with this range of parameters (*p*_*max*_ = 0.95) is shown in Figure 3I. In the figure, as many as twelve repeated firing sequences of length 3 (for the definition of repeated firing sequences, see Figure 2A) are observed. These repeated firing sequences occupy a large fraction of the firing activity in the figure, indicating the emergence of a rich variety of stereotypical firing sequences.

### 2.3. Reproduction of evoked firing sequences and their spontaneous replays

**Figure 4:**
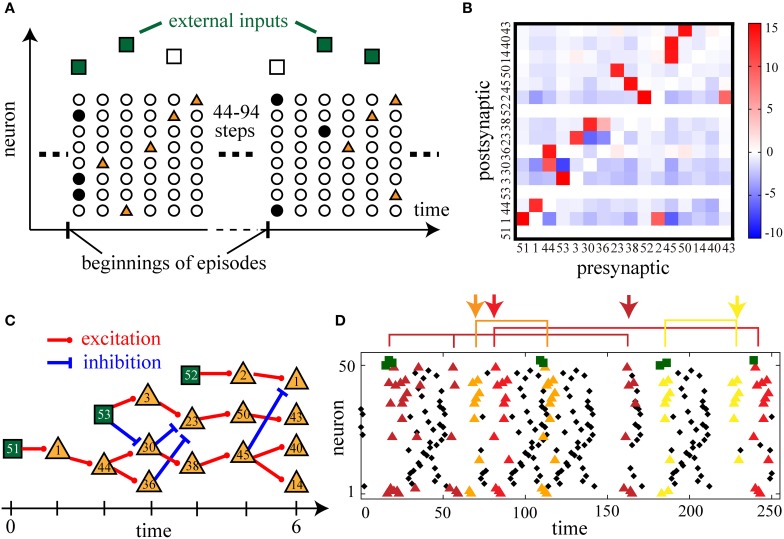
**Sensory-evoked firing sequences and their spontaneous replays are reproduced by applying the learning rule to recurrent neural networks with external inputs**. **(A)** A schematic illustration of the learning of external input sequences. Filled symbols represent the firing of a neuron (vertical axis) at a certain time step (horizontal axis). Once in 50–100 time steps, at the beginning of an input episode, external input neurons fire stochastically in a fixed sequence (dark green squares when fired and empty squares when not fired). In the recurrent network, neurons respond to these inputs (brown triangles). **(B)** A matrix of connection weights between sequence-related neurons. We picked up the sequence-related neurons until the sixth poly-synaptic transmission via strong excitation (*w*_*ij*_ > 8.0) from the firing of the 51st external input neurons. **(C)** A diagram of sequential firing transmission found in the weight matrix in **(B)**. By putting *t* = 0 at the first time step of an external input sequence, possible strong excitation and inhibition are drawn until *t* = 6. **(D)** Typical raster plots after learning. Colored triangles are the firing of those neurons picked up in **(B)**. Each combination of spikes of external input neurons activates a similar but distinct firing sequence indicated by triangles of a different color. Spontaneous replays of partial sequences of the evoked activity are also repeatedly observed (indicated by arrows). The following parameters have been used in this simulation: ϵ = 0.01, *c*_η_ = 2.0, *c*_κ_ = 3.0, *c*_ζ_ = 10.0, *p*_0_ = 0.02, *p*_max_ = 0.98, *N* = 50, τ = 15, *T* = 50000.

In the previous section, we have demonstrated that the present learning rule reproduces repeated sequences in spontaneously firing neural networks. In experimental studies, similarities between firing sequences in spontaneous activity and firing sequences in sensory-evoked activity have been reported (Skaggs and McNaughton, 1996; Lee and Wilson, 2002; Yao et al., 2007). To investigate the relationship between firing sequences in these two types of activity, we further numerically examine the Infomax learning of a neural network driven by external inputs. We prepare a recurrent neural network (RN) consisting of fifty neurons (the 1st–50th neurons), all of which receive feedforward inputs from three external neurons (the 51st–53rd neurons (EXT)). Sensory inputs are modeled by the firing activity of these three external neurons. As represented by the squares in the diagram of Figure 4A, the external neurons fire in a fixed sequence that starts at a random timing with inter-episode intervals uniformly taken from fifty to one hundred time steps. Within the fixed sequences, the 51st neuron fires at the first time step of the sequence, the 53rd neuron at the third time step, and the 52nd neuron at the fifth time step, respectively. Given the fixed timing within the sequences, the external neurons fire stochastically and independently with a probability of 1/2, representing the variability of the environment. As a consequence, the recurrent network receives 2^3^ − 1 = 7 kinds of slightly different input sequences with equal probabilities. Denoting the neurons in the recurrent network as *x*^*t*^_RN_ ∈ {0, 1}^50^ and the external input neurons as *x*^*t*^_EXT_ ∈ {0, 1}^3^, we maximize the mutual information$\text{I}\left[x_{\text{RN}}^{t};x_{\text{RN}}^{t-1},x_{\text{EXT}}^{t-1}\right]$. Although $\text{I}\left[x_{\text{RN}}^{t},x_{\text{EXT}}^{t};x_{\text{RN}}^{t-1},x_{\text{EXT}}^{t-1}\right]$ should be maximized according to the definition of the Infomax learning on recurrent networks, the present learning rule cannot directly maximize this latter mutual information. However, the above two types of mutual information are almost equal if dependence between *x*^*t*^_*EXT*_ and *x*^*t* − 1^_*EXT*_ is small. Hence, we have ignored the small dependence of *x*^*t*^_*EXT*_ on *x*^*t* − *u*^_*EXT*_ (1≤ u≤ 4), maximizing the former information $\text{I}\left[x_{\text{RN}}^{t};x_{\text{RN}}^{t-1},x_{\text{EXT}}^{t-1}\right]$. Then, for the updates of both the recurrent connections and the feedforward connections from the external neurons in Equations (3) and (4), the index *i* runs through the neuron indices of RN, while the index *j* runs through those of both RN and EXT. Initially, we set three particular connections from the external neurons to be a large value (*w*_1, 51_, *w*_2, 52_, *w*_3, 53_ = 100) for the modeling of an established pathway of sensory signals, and the other connections to be small values (randomly taken from [−0.1, 0.1]). Then, we update both the recurrent and feedforward connections according to the present learning rule.

After learning, we observe that the network apparently displays several stereotypical firing sequences in response to the input sequences. Since these firing sequences are thought to be organized by sparsely distributed strong connections similarly to the cases in the previous sections, we analyze the connectivity matrix in detail. We pick up sequence-related neurons activated by poly-synaptic transmission due to strong excitatory connections (*w*_*ij*_ > 8.0) until the sixth transmission from the firing of the 51st neuron, and construct a weight matrix of the connectivity among the picked-up neurons (Figure 4B). In this weight matrix, we find sequential activation and inhibition due to the strong connections (Figure 4C) that are thought to underlie the input-evoked stereotypical firing sequences (colored triangles in Figure 4D). To be more precise, we find that three chains of strong excitatory transmission originate from either one of the three external neurons and have crosstalk with each other via strong excitatory and inhibitory transmission. Each of the seven combinations of input firing consequently activates a distinct part of the transmission cascade in Figure 4C, and results in a similar but distinct firing sequence indicated by different colors of triangles in Figure 4D. For example, when all the three external neurons fire, the evoked firing sequence is [51]→ [1]→ [53, 44]→ [3, 36]→ [52, 23]→ [2, 50]→ [1, 43] (dark brown triangles) in the order of time passage while some neurons fail to fire occasionally. This is similar to but different from the sequence activated by the combination of the 51st and 52nd neurons, [51]→ [1]→ [44]→ [30, 36]→ [52, 38]→ [2, 45]→ [14, 40] (light yellow triangles). Furthermore, such sequences are repeatedly activated in spontaneous firing between the input episodes as indicated by the arrows of different colors in Figure 4D. Thus, the present learning rule has reproduced replays of sensory-evoked firing sequences.

### 2.4. Reproduction of simple-cell-like receptive field properties

**Figure 5:**
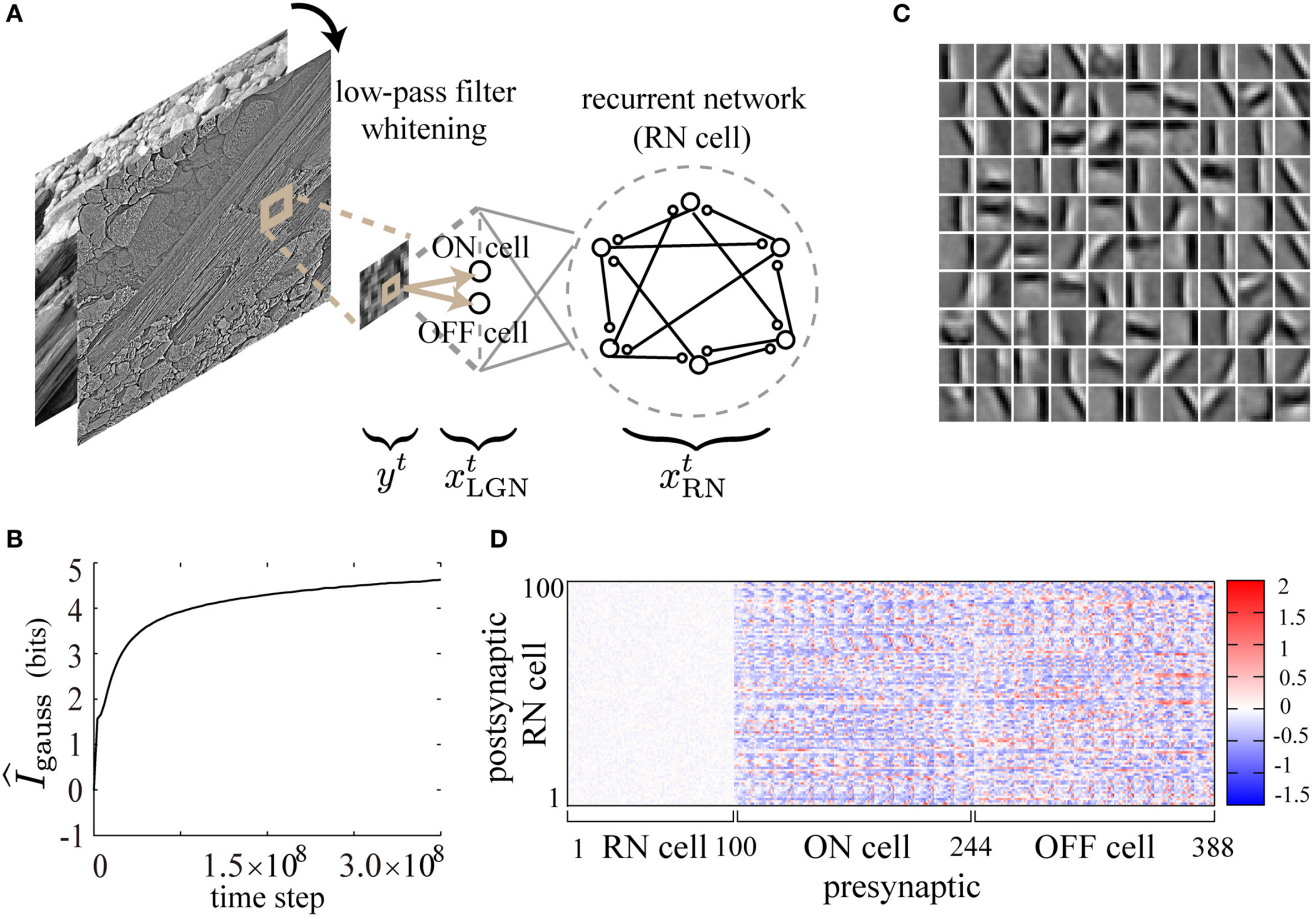
**Feature extraction from natural images by the present learning rule**. **(A)** Randomly taken 12× 12-pixel patches from gray-scaled, low-pass filtered and whitened natural images are used as inputs to a recurrent neural network through ON and OFF cells. **(B)** $\hat{I}$_*gauss*_ is calculated during the course of learning. **(C)** STAs of input images are displayed as 10× 10 small image patches in a gray-scale (1: black, 255: white), representing the receptive field properties of the neurons in the recurrent network after learning. Each small patch corresponds to an STA due to the firing of a neuron in the recurrent network. **(D)** The connection weight matrix after learning is displayed on a color scale. The following parameters have been used in this simulation: ϵ = 0.02, *c*_η_ = 250.0, *c*_κ_ = 150.0, *c*_ζ_ = 1000.0, *p*_0_ = 0.0015, *p*_max_ = 0.95, *N* = 100, τ = 5, *T* = 50000.

It is well known that Infomax learning is mathematically equivalent to independent component analysis (ICA) in the limit of small noise, and results in the extraction of filters similar to orientation selectivity of simple cells in V1 when it is applied to the analysis of natural images (Bell and Sejnowski, 1995, 1997). In this section, we examine numerically whether the present learning rule also achieves such feature extraction. As described in Figure 5A, a 12 × 12 pixel patch *y*^*t*^ taken in the independently and identically distributed (i.i.d.) manner from low-pass filtered, gray-scaled and whitened natural images (Olshausen and Field, 1997) is used as an input at each time step. Similar preprocessing of input images is thought to be done in the retina and the lateral geniculate nucleus (LGN) before the processing in V1 (Atick, 1992). Following Tanaka et al. (2009), as a model of LGN, we prepare a pair of ON and OFF neurons, *x*^*t*^_*i*, ON_ and *x*^*t*^_*i*, OFF_, for each pixel *y*^*t*^_*i*_ (1 ≤ *i* ≤ 144), which fire with the following probabilities proportional to positive and negative intensities of the pixel, respectively:

$$\begin{array}{l} \{\begin{array}{l} p\left(x_{i,\text{ON}}^{t}=1|y^{t}\right)=\min\left\{\xi y_{i}^{t},1\right\}\\ p\left(x_{i,\text{OFF}}^{t}=1|y^{t}\right)=0 \end{array}\text{ for }y_{i}^{t}\geq 0,\\ \{\begin{array}{l} p\left(x_{i,\text{ON}}^{t}=1|y^{t}\right)=0\\ p\left(x_{i,\text{OFF}}^{t}=1|y^{t}\right)=\min\left\{-\xi y_{i}^{t},1\right\} \end{array}\text{ for }y_{i}^{t}\leq 0. \end{array}$$

The constant ξ is set to be 1.65 so that the mean firing rate *p*_input_ of ON- and OFF-neurons is around 0.15. Each of those input neurons feeds synaptic inputs to all the neurons *x*^*t*^_*i*, RN_ (1 ≤ *i* ≤ 100) in the recurrent network (RN).

Then, we apply the present learning rule to this model for the maximization of I [*x*^*t*^; *x*^*t* − 1^], denoting ${\left(x^{t}\right)}^{\prime }=\left\{{\left(x_{\text{LGN}}^{t}\right)}^{\prime },{\left(x_{\text{RN}}^{t}\right)}^{\prime }\right\}$ and ${\left(x_{\text{LGN}}^{t}\right)}^{\prime }=\left\{{\left(x_{\text{ON}}^{t}\right)}^{\prime },{\left(x_{\text{OFF}}^{t}\right)}^{\prime }\right\}$ (the prime symbols represent transposition of vectors). We further obtain I [*x*^*t*^; *x*^*t* − 1^] = I [*x*^*t*^_RN_; *x*^*t* − 1^] because of the independence between *x*^*t*^_LGN_ and *x*^*t*^_RN_, as well as the independence between *x*^*t*^_LGN_ and *x*^*t* − 1^_LGN_ (recall that *x*^*t*^_LGN_ is generated in an i.i.d. manner from the natural images at each time step). Thus, we maximize I [*x*^*t*^_RN_; *x*^*t* − 1^] instead of I [*x*^*t*^; *x*^*t* − 1^], calculating γ^*t*^_*k*_ with respect to only *x*^*t*^_RN_ in Equations (3) and (4). For the updates of both the recurrent connections and the feedforward connections from LGN in Equations (3) and (4), the index *i* runs through the neuron indices of RN, while the index *j* runs through those of both RN and LGN.

As shown in Figure 5B, $\hat{I}$_*gauss*_ increases monotonically and tends to saturate during the course of learning, suggesting that the mutual information is successfully maximized. After learning, we calculate reverse correlations by taking spike-triggered average (STA) of stimulus images (see Methods and Ringach and Shapley, 2004 for details), in order to examine the response properties of the RN neurons. As shown in STAs of Figure 5C, simple-cell-like orientation selectivity is reproduced in the simulation with a low firing rate (*p*_0_ = 0.0015) of the neurons in the recurrent network and a relatively high firing rate (*p*_input_ = 0.15) of the input neurons. The neurons acquire selectivity to edge-shaped (Gabor-function-like) contrasts of various positions, sizes, phases, frequencies, and orientations as observed in macaque V1 (Ringach, 2002). As shown in the weight matrix in Figure 5D, the synaptic weights of the recurrent connections after learning are almost zero, probably reflecting the i.i.d. presentation of the input images. In conclusion, our biologically plausible learning rule reproduces orientation selectivity of simple cells in V1 in a similar way to the previous Infomax algorithms on feedforward and recurrent neural networks (Bell and Sejnowski, 1997; Tanaka et al., 2009).

## 3. Discussion

In this paper, we have constructed a biologically plausible rule for the recurrent Infomax learning proposed in Tanaka et al. (2009), and reproduced the following several firing profiles of cortical neurons: (1) cell-assembly-like repeats of precise firing sequences, (2) neuronal avalanches, (3) replays of sensory-evoked firing sequences, and (4) orientation selectivity of simple cells in V1.

### 3.1. Biologically plausible learning rule and its similarity to STDP

In the preceding study (Tanaka et al., 2009), the learning algorithm for the recurrent Infomax was biologically implausible, which might be due to a common difficulty in considering biological realization of learning algorithms in recurrent neural networks. Actually, until recently, most learning rules proposed as biologically plausible have been limited mainly to feedforward neural networks (Savin et al., 2010; Zylberberg et al., 2011; Tanaka et al., 2012). In the present study, we have constructed a learning rule for the recurrent Infomax, according to which weight changes are computed by each postsynaptic neuron through its local interactions with other neurons and modulation by global signals γ^*t*^_*k*_ (1 ≤ *k* ≤ 4). Although the present learning rule requires the global signals γ^*t*^_*k*_, we presume neural substrates for these global signals as follows. We first note that γ^*t*^_1_ − γ^*t*^_3_ − γ^*t*^_4_ is a simple population sum of non-linearly transformed activities of single neurons. On the other hand, γ^*t*^_2_ is a non-linear function of the population activity *m*^*t*^ = ∑_*i*_*x*^*t*^_*i*_ and its temporal integration 〈 *m*^*t*^〉_*T*_. Thus, we should consider two different neural substrates.

For the calculation of γ^*t*^_1_ − γ^*t*^_3_ − γ^*t*^_4_, before the summation over the neuronal population is taken, a complicated non-linear function of neural activity *x*^*t*^_*i*_ and *s*^*t*^_*i*_ must be computed by each of the neurons. It would be reasonable to assume that such complicated computations are realized through intracellular processes. Then, as a neural substrate for the simple summation of these intracellularly computed quantities over the neuronal population, we are able to assume a rapidly diffusing substance (for example, nitric oxide, neuropeptides, lipid metabolites, endcannabinoids, and so on) emitted by each neuron with an amount proportional to the intracellularly computed quantity. As a consequence of its rapid diffusion, its concentration in the neuronal circuit represents the population sum, that is, γ^*t*^_1_ − γ^*t*^_3_ − γ^*t*^_4_. The multiplication of ${\langle \psi_{i}^{t}x_{j}^{t}\rangle }_{\tau }$ by γ^*t*^_1_ − γ^*t*^_3_ − γ^*t*^_4_ may then be realized through the modulation of intracellular signaling in the target neuron *i* by the rapidly diffusing substance. On the other hand, for the calculation of γ^*t*^_2_, the population activity *m*^*t*^ and its temporal integration 〈 *m*^*t*^〉_*T*_ need to be non-linearly transformed. We suggest interneurons, for example, as a neural substrate for this transformation. Some class of interneurons receives massive inputs from surrounding excitatory neurons and returns feedback to them (Markram et al., 2004). Thus, these interneurons may possibly compute the non-linear function of *m*^*t*^ and 〈 *m*^*t*^〉_*T*_ in γ^*t*^_2_, and then amplify ${\langle \psi_{i}^{t}x_{j}^{t}\rangle }_{\tau }$ via intracellular signaling in the target neuron *i* by acting on the metabotropic receptors.

Hence, as we have discussed, all the processes included in Equations (3) and (4) of the present learning rule are biologically plausible in principle. It is worth noting that the above computational discussion may provides a novel perspective on the roles played by many kinds of neurotransmitters and neuromodulators and many types of inhibitory interneurons. Since the present learning rule has successfully reproduced several characteristic firing profiles of cortical neurons, we suggest the importance of experimental investigation in the candidate neural substrates for the present learning rule.

We further note similarities between the local quantity ψ^*t*^_*i*_*x*^*t*^_*j*_ in the present learning rule and synaptic changes according to classical STDP (Bi and Poo, 1998). For γ^*t*^_*k*_ > 0, ψ^*t* − *u*^_*i*_ *x*^*t* − *u*^_*j*_ (*u* ≥ 1) in the present learning rule represents the potentiation of synaptic weights at time *t* when the firing of the presynaptic neuron at time *t* − *u* precedes the firing of the postsynaptic neuron at time *t* − *u* + 1 (the red lines in Figure 1). On the other hand, when the postsynaptic neuron fails to fire after the firing of the presynaptic neuron, the synapse is depressed (the blue lines in Figure 1). When synaptic changes according to 〈 ψ^*t*^_*i*_*x*^*t*^_*j*_〉_τ_ with γ^*t*^_*k*_ > 0 are measured in the same setting as classical STDP (Bi and Poo, 1998), the synaptic changes would fall on a function of relative spike timing similar to that of classical STDP. Therefore, we suggest STDP as a candidate neural substrate for the computation of ψ^*t*^_*i*_*x*^*t*^_*j*_. In addition, the present learning rule has the same mathematical structure as the reward-modulated STDP recently developed for the neural implementation of policy-gradient algorithms in reinforcement learning (Florian, 2007; Frémaux et al., 2013). From this viewpoint, ψ^*t*^_*i*_*x*^*t*^_*j*_ and γ^*t*^_*k*_ in the present learning rule corresponds to STDP and reward/temporal-difference (TD) error signals, respectively. The reward-modulated STDP was constructed using continuous-time neuron models, and we reasonably expect that the present learning rule can be extended to a learning rule for continuous time models by a similar derivation. In the present study, however, we have taken advantage of the mathematical clarity and computational tractability of discrete-time models. We additionally note that, even if we derive an extension to a continuous time model, there will remain arbitrariness in the determination of Δ *t* of the corresponding objective function I [*x*^*t*^; *x*^*t* − Δ *t*^]. In other words, a kind of coarse-graining that determines the time scale of encoding Δ *t* will be essential.

As discussed above, the present learning rule is biologically plausible in principle, but it is necessary to note the following points to consider further correspondences between the present model and real cortical circuits. Firstly, in our model, single neurons can make both excitatory and inhibitory synapses, violating Dale's law. In order to settle this issue, we may consider the highly non-random connectivity among excitatory and inhibitory neurons in the cerebral cortex. The cerebral cortex has columnar structures, in which neurons with similar responsiveness are aligned in vertical strips. Thus, it would be reasonable to expect that there is a pair of excitatory and inhibitory neurons with the same responsiveness. For example, we assume that there is a local strong constraint of the internal variable *s*^*t*^_*i*_ to be the same value between the pair. Then, this pair plays the same role as the idealized single neuron in our model. Secondly, our discussion is limited to learning in local microcircuits, not to learning in wider brain regions such as the whole cerebral cortex, as we have assumed locally diffusing or cellular substrates for the calculation of the global signals γ^*t*^_*k*_. This contrasts with dopamine as a global signal for the modulation of STDP in reinforcement learning (Florian, 2007; Frémaux et al., 2013). In the latter case, we can assume that dopamine is released at the same time in very wide brain regions. In the future extension of our model, on the analogy of the case of dopamine, we may consider similar monoaminergic mechanisms for the integration of local microcircuits. Thirdly, the present learning rule is not in the form of the association of firing pairs in the case of negative ψ^*t*^_*i*_*x*^*t*^_*j*_, which is different from the concept of classical STDP. This discrepancy is also common to the reward-modulated STDP (Florian, 2007), but a heuristic approximation has been proposed for the explanation of the associative LTD part of classical STDP in Florian (2007). In contrast, we have not incorporated such heuristics into the present rule so as to clearly reproduce the firing profiles of cortical neurons. Considering that the present learning rule has successfully reproduced the firing profiles observed in real cortical circuits, and that classical STDP was measured in rather artificial experimental conditions, we suggest the importance of experimental investigation for the precise form of the plasticity in physiological conditions. In this context, several different forms of STDP have recently been reported (Caporale and Dan, 2008). Actually, non-associative synaptic changes similar to negative ψ^*t*^_*i*_*x*^*t*^_*j*_ in the present learning rule have recently been reported in the neural system of the electric fish (Han et al., 2000). It should further be noted that STDP is only one aspect of more complicated intracellular processes including intracellular calcium dynamics, and some researchers have explored models of these processes behind STDP (Bienenstock et al., 1982; Shouval et al., 2002; Toyoizumi et al., 2005). We intend to consider the consistency between the plasticity of real cortical neurons and analytically derived learning rules in future studies.

### 3.2. Reproduction of repeated firing sequences and neuronal avalanches

There have been many computational studies on sequential firing transmission, such as precise firing sequences and neuronal avalanches, since the age of the theory of cell assembly and synfire-chain (Hebb, 1949; Abeles, 1991). Several studies have demonstrated that certain specialized network structures allow reliable sequential firing transmission (Diesmann et al., 1999; Teramae and Fukai, 2007; Teramae et al., 2012). From the viewpoint of learning, an author has numerically demonstrated that stereotypical sequences of firing are generated by STDP in recurrent neural networks and represent memory of input stimulation (Izhikevich, 2006). Several issues, however, have remained to be settled, concerning the instability of the firing sequences under the reorganization according to their learning rule and the lack of theoretical interpretability of STDP. The instability and the lack of interpretability of STDP in this context has been recognized, and a great effort has been made on the study of neural mechanisms for the stabilization of spontaneous activity self-organized by STDP (Fiete et al., 2010; Gilson and Fukai, 2011). Our approach solves this problem by realizing stable self-organization of spontaneous activity through the modulation of STDP by the global signals in the present learning rule, if we admit that STDP is the neural substrate for ψ^*t*^_*i*_*x*^*t*^_*j*_ in Equation (3).

In the self-organization of spontaneous activity, the present learning rule has reproduced neuronal avalanches for small *p*_0_ and *p*_max_ values, and repeats of precise firing sequences for larger *p*_max_ values. A computational study has recently reported that recurrent neural networks with sparsely distributed strong connections display apparently asynchronous and irregular firing activity consisting of precise sequential firing transmission (Teramae et al., 2012). Such a network structure is supported by recent experimental results that magnitudes of synaptic potentials in the cerebral cortex have a skewed distribution, and only a small fraction of the synapses evokes very large excitatory postsynaptic potentials (Feldmeyer et al., 2002; Song et al., 2005). There is more supporting evidence that the firing activity of cortical neurons in behaving animals is apparently asynchronous and irregular (Renart et al., 2010). The present results are consistent with all of these previous computational and experimental results, since the firing activity with many repeated sequences in the present study is apparently asynchronous and irregular, and the underlying network structure consists of sparsely distributed strong connections. Thus, the present learning rule provides a candidate learning mechanism for the previous experimental results.

### 3.3. Reproduction of replays of sensory-evoked firing sequences

We have further applied the learning rule not only to spontaneously firing neural networks but also to neural networks with external inputs. We have observed that stochastically varying external inputs reliably evoked stereotypical firing sequences in the recurrent network after learning. In the evoked activity, stochastic variation of external inputs was encoded into the activation of distinct parts of a common network structure. Such encoding of similar but distinct external inputs into a common network structure is thought to be more efficient than encoding them into different network structures. Such an efficient encoding would be preferable for limited resources in animal brains and under environmental uncertainty in the real world. Furthermore, we have found that the evoked sequences were replayed spontaneously. These results suggest that the present learning rule is a candidate learning mechanism for the emergence of sensory-evoked firing sequences and its replays in the sleeping state or in the quietly awake state (Skaggs and McNaughton, 1996; Lee and Wilson, 2002; Yao et al., 2007). Since sleep is considered to play a role in the consolidation of memory (Stickgold and Walker, 2005), the present learning rule might provide a clue to the underlying mechanisms of memory consolidation in future studies.

### 3.4. Reproduction of orientation selectivity in V1

For feedforward neural networks, several biologically plausible learning rules have been proposed to explain the emergence of orientation selectivity in V1 (Zylberberg et al., 2011; Tanaka et al., 2012). These learning rules are, however, not applicable to the dynamics of recurrent neural networks. Several computational studies have proposed STDP as a learning mechanism for independent component analysis (ICA) in the brain, and accounted for the emergence of orientation selectivity (Savin et al., 2010; Gilson et al., 2012). However, the demonstration of the performance of their learning rules has been limited to the extraction of relatively small numbers (up to 10) of features. In the context of unsupervised learning by means of STDP, Nessler et al. (2013) theoretically showed that STDP provides a mechanism for Bayesian computation and demonstrated unsupervised clustering with their learning rule, but their theory did not explain the emergence of orientation selectivity.

The present Infomax learning rule has an STDP-like component as discussed in the previous section, and Infomax learning is considered to be almost equivalent to ICA in many situations. Although the previous studies of STDP-based learning rules (Savin et al., 2010; Gilson et al., 2012) have suggested that STDP provides a learning mechanism for ICA, the present learning rule has clearly outperformed their STDP-based learning rules by reproducing one hundred simple-cell-like receptive fields of various orientations, phases, positions, and sizes, compared with the up-to-ten receptive fields reproduced in the previous studies. The difference of the present learning rule from the other STDP-based learning rules for ICA or clustering (Savin et al., 2010; Gilson et al., 2012; Nessler et al., 2013) is the correlation term *A*_2_ in Equation (2), which has been introduced for the active decorrelation of firing activity. Thus, it is expected that *A*_2_ has played a role in further penalizing residual dependence between neuronal firing, suggesting that a mechanism for active decorrelation such as *A*_2_ in addition to STDP is effective for ICA. It is noticeable that *A*_2_ also represents population sparseness, since it is a long-time average of the square of population activity. This provides an intuitive explanation of the reason for which learning according to both the Infomax and sparse coding principles leads to the extraction of similar features from natural images (Bell and Sejnowski, 1997; Olshausen and Field, 1997).

## 4. Methods

### 4.1. Construction of the approximate objective function

In this section, we construct the objective function, Equation (2), that approximates Equation (1), and then derive the learning rule, Equations (3) and (4). First, the mutual information is decomposed as

(8) $$\begin{array}{l} \text{I}\left[x^{t};x^{t-1}\right]=\text{H}\left[x^{t}\right]-\text{H}\left[x^{t}|x^{t-1}\right]\\ \;=\text{H}\left[x^{t}\right]-\sum_{i}\text{H}\left[x_{i}^{t}|x^{t-1}\right]\\ \;\leq \sum_{i}\text{H}\left[x_{i}^{t}\right]-\sum_{i}\text{H}\left[x_{i}^{t}|x^{t-1}\right]\\ \;=\sum_{i}\text{I}\left[x_{i}^{t}|x^{t}\right]\\ \;=\tilde{A_{1}} \end{array}$$

The second equality is due to the conditional independence of the dynamics. The third equality holds and the mutual information is decomposed if and only if {*x*^*t*^_*i*_}_1 ≤ *i* ≤ *N*_ are independent. Then, we approximate the mutual information with $\tilde{A_{1}}$ of Equation (8) by subtracting a penalty term so as to bound the firing distribution near the independent distribution. Given that *p*_0_ ≪ 1 and positive second-order correlations ${\langle x_{i}^{t}x_{j}^{t}\rangle }_{\infty }-{\langle x_{i\text{​​​}}^{t}\rangle }_{\infty }{\langle x_{j}^{t}\rangle }_{\infty }$ are *O*(*p*^2^_0_) for *i* ≠ *j*, negative second-order correlations and all multi-body correlations are necessarily *O*(*p*^2^_0_). So, ignoring the negative and multi-body correlations, we use $A_{2}=\kappa \sum_{i<j}\left\{{\langle x_{i}^{t}x_{j}^{t}\rangle }_{\infty }-{\langle x_{i}^{t}\rangle }_{\infty }{\langle x_{j}^{t}\rangle }_{\infty }\right\}$, the sum of second-order correlations as a penalty term. We further subtract the firing-rate term $A_{3}=\frac{\eta }{2}\sum_{i}{\left({\langle x_{i}^{t}\rangle }_{\infty }-p_{0}\right)}^{2}$ for the constraint of average firing rates to be *p*_0_, and the fluctuation term $A_{4}=\frac{\zeta }{2}\sum_{i}{\langle {\left(s_{i}^{t}-s_{0}\right)}^{2}\rangle }_{\infty }$ for the confinement of fluctuation of internal variables *s*^*t*^_*i*_ within a physiologically reasonable range. Hence, we obtain an objective function $\tilde{A_{1}}$ − *A*_2_ − *A*_3_ − *A*_4_. In preliminary simulations, however, we have found that the derivatives of this objective function are very small over a wide range of parameters where $\tilde{A_{1}}$ is relatively small, and that learning does not actually proceed (data not shown). We therefore take the logarithm of I [*x*^*t*^_*i*_|*x*^*t* − 1^] in $\tilde{A_{1}}$ in order to accelerate learning. It provides a lower bound of $\tilde{A_{1}}$, since $\log\frac{1}{N}\sum_{i}\text{I}\left[x_{i}^{t};x^{t-1}\right]\geq \frac{1}{N}\sum_{i}\log\text{I}\left[x_{i}^{t};x^{t-1}\right]$ (Jensen inequality). Maximization of this lower bound implies maximization of $\tilde{A_{1}}$ if I [*x*^*t*^_*i*_; *x*^*t* − 1^] are equal for 1 ≤ *i* ≤ *N*, which is almost the case in the present simulation. In this way, we obtain and restate the objective function (Equation (2)):

(9) $$\begin{array}{l} A=\underset{A_{1}}{\underset{︸}{\sum_{i}\log\text{I}\left[x_{i}^{t};x^{t-1}\right]}}-\underset{A_{2}}{\underset{︸}{\kappa \sum_{i<j}\left({\langle x_{i}^{t}x_{j}^{t}\rangle }_{\infty }-{\langle x_{i}^{t}\rangle }_{\infty }{\langle x_{j}^{t}\rangle }_{\infty }\right)}}\\ \;-\underset{A_{3}}{\underset{︸}{\frac{\eta }{2}\sum_{i}{\left({\langle x_{i}^{t}\rangle }_{\infty }-p_{0}\right)}^{2}}}-\underset{A_{4}}{\underset{︸}{\frac{\zeta }{2}\sum_{i}\langle {\left(s_{i}^{t}-s_{0}\right)}^{2}\rangle }}. \end{array}$$

It might also be naturally considered to use *A*′_1_ in the following equation for the acceleration of learning, taking the logarithm outside the sum of neuron-wise information terms, rather than using the lower bound *A*_1_.

(10) $$\begin{array}{l} A^{\prime }=\underset{A_{1}^{\prime }}{\underset{︸}{N\log\sum_{i}\text{I}\left[x_{i}^{t};x^{t-1}\right]}}-\underset{A_{2}}{\underset{︸}{\kappa \sum_{i<j}\left({\langle x_{i}^{t}x_{j}^{t}\rangle }_{\infty }-{\langle x_{i}^{t}\rangle }_{\infty }{\langle x_{j}^{t}\rangle }_{\infty }\right)}}\\ \;-\underset{A_{3}}{\underset{︸}{\frac{\eta }{2}\sum_{i}{\left({\langle x_{i}^{t}\rangle }_{\infty }-p_{0}\right)}^{2}}}-\underset{A_{4}}{\underset{︸}{\frac{\zeta }{2}\sum_{i}\langle {\left(s_{i}^{t}-s_{0}\right)}^{2}\rangle }}. \end{array}$$

In this case, we can also derive a learning rule which is biologically plausible in the same sense as Equations (3) and (4), by replacing γ^*t*^_1_ with

(11) $$\gamma_{1}^{\prime ,t}=\frac{1}{\sum_{i}{\langle \log\frac{p\left(x_{i}^{t}|x^{t-1}\right)}{Z_{i}^{t}}\rangle }_{T}}\sum_{i}\log\frac{p\left(x_{i}^{t}|x^{t-1}\right)}{Z_{i}^{t}}.$$

**Figure 6:**
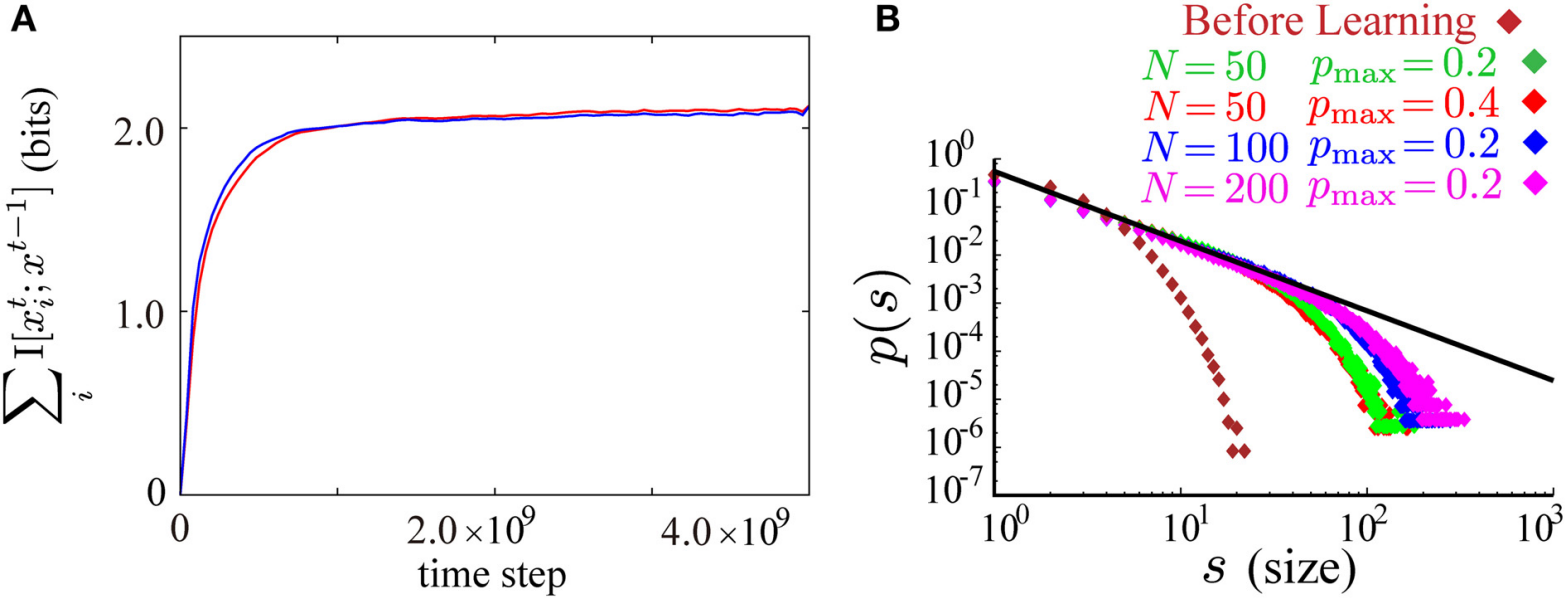
**(A)** Time evolution of the sum of the neuron-wise information terms ∑_*i*_I [*x*^*t*^_*i*_; *x*^*t* − 1^] during the course of learning according to the objective function with the lower bound *A*_1_, Equation (9) (the red curve), and the modified objective function obtained by replacing *A*_1_ with *A*′_1_, Equation (10) (the blue curve). **(B)** The results in Figure 3B are reproduced after learning with the modified objective function and the same learning parameters as in Figure 3B. Log-log plots of size of bursts *s* against the occurrence of bursts of that size *p*(*s*) before (brown) and after learning (red for *p*_max_ = 0.4 and *N* = 50, green for *p*_max_ = 0.2 and *N* = 50, blue for *p*_max_ = 0.2 and *N* = 100, and magenta for *p*_max_ = 0.2 and *N* = 200). The size distributions after learning fall on the straight line of slope around −3/2 (the black line).

In the two learning processes according to either of *A*_1_ or *A*′_1_, the values of the sum of neuron-wise information terms ∑_*i*_I [*x*^*t*^_*i*_; *x*^*t* − 1^] are almost equal during the course of learning (Figure 6A). In simulations with *A*′_1_, the network actually learns to reproduce almost the same results as in the main text of this paper. For example, the same power law as in Figure 3B is reproduced after learning according to the objective function *A* ′ (Figure 6B). The learning rule derived from *A*′, however, requires three different neural substrates for the global signals. Inspecting γ′^, *t*^_1_, we notice that it is necessary to divide a global term ∑_*i*_log *p*(*x*^*t*^_*i*_; *x*^*t* − 1^)/*Z*^*t*^_*i*_ by another global term ∑_*i*_ 〈 log *p*(*x*^*t*^_*i*_|*x*^*t* − 1^)/*Z*^*t*^_*i*_〉_*T*_. Hence, we cannot decompose γ′^, *t*^_1_ into neuron-wise terms, and must consider two different neural substrates for this term. We considered that the assumption of more global signals reduces biological plausibility. Thus, we do not adopt *A*′_1_ in the present study.

**Figure 7:**
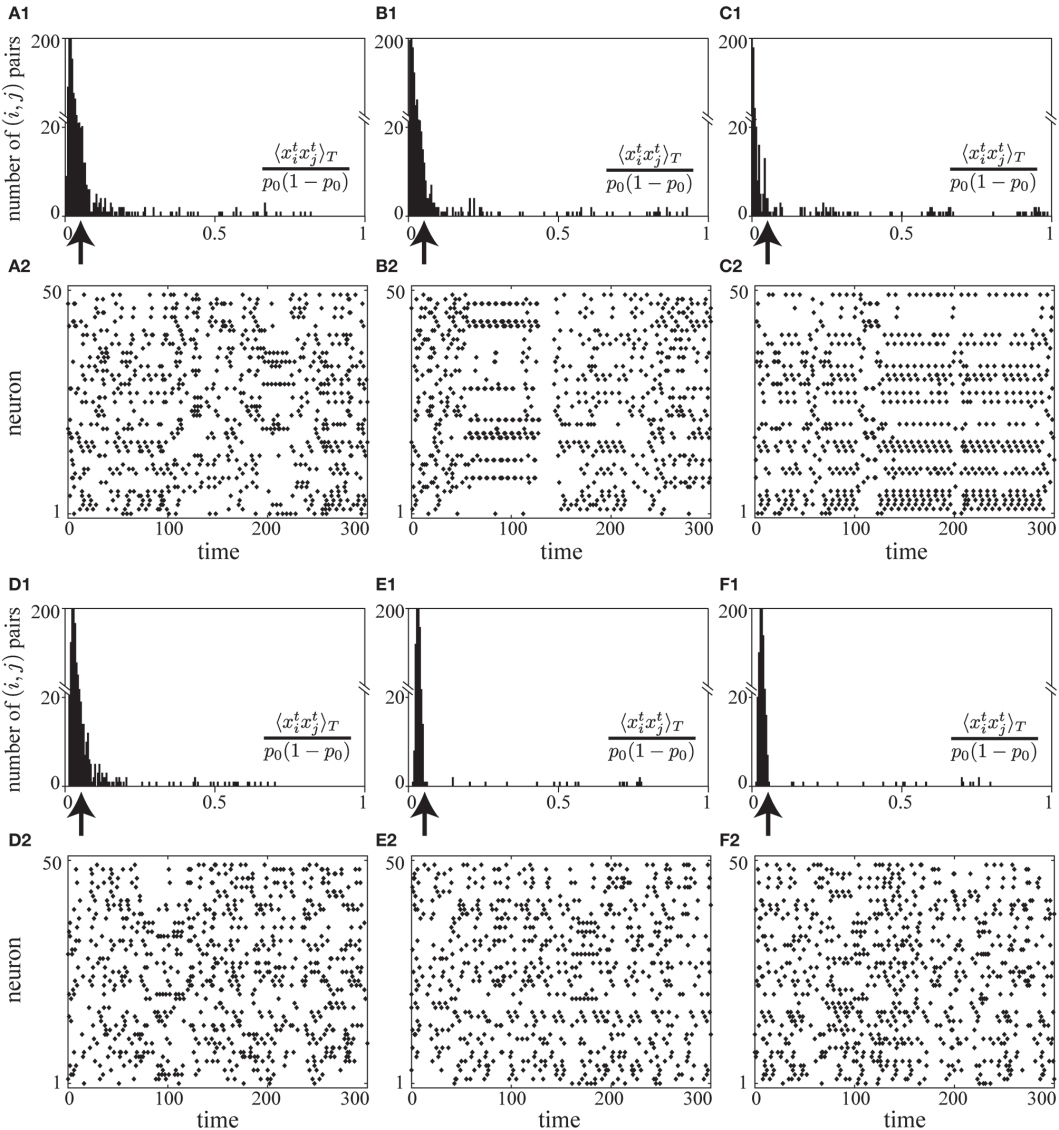
**(A1–C2)** A simulation of learning in a spontaneously firing network without the fluctuation term *A*_4_. Histograms of pairwise correlations 〈 *x*_*i*_*x*_*j*_〉_*T*_/{*p*_0_(1 − *p*_0_)} (*i* ≠ *j*) **(A1–C1)** and raster plots **(A2–C2)** at different time steps during learning (the 5× 10^8^, 1× 10^9^, and 2× 10^9^-th steps, respectively) are shown. The learning coefficient *c*_ζ_ is set to be 10000.0 so that the fluctuation term *A*_4_ does not take effect. **(D1–F2)** A simulation of learning in a spontaneously firing network under the effects of the fluctuation term *A*_4_. Histograms of pairwise correlations 〈 *x*_*i*_*x*_*j*_〉_*T*_/{*p*_0_(1 − *p*_0_)} (*i* ≠ *j*) **(D1–F1)** and raster plots **(D2–F2)** at different time steps during learning (the 5× 10^8^, 1× 10^9^, and 2× 10^9^-th steps, respectively) are shown. The learning coefficient *c*_ζ_ is set to be 3.0, the same value as that used in the simulation in Figure 2. In **(A1–C1)** and **(D1–F1)**, the arrows indicate the value of correlation between the independent firing of *x*^*t*^_*i*_ and *x*^*t*^_*j*_. For the simulation in this figure, the same learning coefficients as those used in Figure 2 are used except *c*_ζ_: ϵ = 0.006, *c*_η_ = 1.5, *c*_κ_ = 1.0, *c*_ζ_ = 3.0, or 10000.0, *p*_0_ = 0.05, *p*_max_ = 0.95, *N* = 50, τ = 15, *T* = 50000.

In a preliminary simulation, we observed that the correlation term *A*_2_ and the firing-rate term *A*_3_ successfully decorrelate neuronal activity and control mean firing rates to be *p*_0_ (see Figures 2D,E). Without the fluctuation term *A*_4_, we found that the learning proceeds successfully in the early stages (the raster plot in Figure 7A2), but overly strong positive and negative synaptic weights form nearly periodic firing patterns at later stages (the raster plots in Figures 7B2,C2). In this nearly periodic pattern of firing, the first term of Equation (8), the entropy term, would be small and thus the mutual information would not be maximized. The origin of this failure is attributed to the fact that the negative and higher-order correlations are no longer negligible in this nearly periodic firing pattern caused by the strong positive and negative connections. In fact, the many negative pairwise correlations near −*p*^2^_0_ balance the large positive pairwise correlations in *A*_2_ as shown in the histograms of correlations in Figures 7B1,C1, thus violating the assumption on which we have constructed *A*_2_. In contrast, we have found that the objective function with the fluctuation term *A*_4_ stably decorrelates firing activity (histograms of correlations in Figures 7D1–F1 and raster plots in Figures 7D2–F2).

In addition, we represent the coefficients of the penalty terms in Equation (9) with the scaled parameters. Assuming that positive second-order correlations and deviations of average firing rates from *p*_0_ are *O*(*p*^2^_0_), we obtain *A*_2_∝ *p*^4^_0_ and *A*_3_∝ *p*^2^_0_. From the numbers of terms in the summations with respect to *i*, *j* in Equation (9), we obtain that *A*_1_ ∝ *N*, *A*_2_ ∝ *N*, *A*_3_ ∝ *N*^2^, and *A*_4_ ∝ *N*. Thus, we have determined the scale as:

(12) $$\kappa =\frac{2}{(N-1)c_{\kappa }p_{0}^{2}},\;\eta =\frac{1}{c_{\eta }^{2}p_{0}^{4}},\;\zeta =\frac{1}{c_{\zeta }^{2}}.$$

### 4.2. Derivation of the biologically plausible learning rule

Before deriving the learning rule, Equations (3) and (4), we first consider the maximization of a general objective function 〈 γ^*t*^〉_∞_ with respect to model parameters *w*_*ij*_ and *h*_*i*_. Here, γ^*t*^ is an arbitrary function of firing patterns *x*^*t*^ and *x*^*t* − 1^, and model parameters, *w*_*ij*_ and *h*_*i*_. More precisely, our aim is to calculate gradients of the following objective function for the gradient ascent method:

$${\langle \gamma^{t}\rangle }_{\infty }=\sum_{\left\{x^{t-1},x^{t}\right\}}p_{s}\left(x^{t-1}\right)p\left(x^{t}|x^{t-1}\right)\gamma^{t}.$$

In the above equation, the summation has been taken over all possible firing patterns for *x*^*t* − 1^ and *x*^*t*^ in {0, 1}^2*N*^. When we try to take gradients of the above quantity, we find that differentiation of the stationary distribution *p*_*s*_(*x*^*t* − 1^) is apparently intractable while differentiation of the other components is easily computed. We notice, however, that we do not need to differentiate the stationary distribution *p*_*s*_(*x*^*t*^) explicitly, assuming that the firing distribution *p*(*x*^*t*^) converges to a unique stationary distribution as time passes, and that the stationary distribution is a smooth function of the model parameters, *w*_*ij*_ and *h*_*i*_. On these assumptions, small changes of *p*_*s*_(*x*^*t* − τ^) for τ ≫ 1 in the objective function eventually vanish at *t* and *t* − 1, and thus the terms including the derivatives of *p*_*s*_(*x*^*t* − τ^) are negligible (see also Baxter and Bartlett, 1999). Then, we can compute the gradients as follows:

(13) $$\begin{array}{l} \frac{\partial }{\partial w_{ij}}{\langle \gamma^{t}\rangle }_{\infty }\\ =\underset{\tau \;\to \;\infty }{\lim}\sum_{\left\{x^{t-\tau },x^{t-\tau +1},\cdots ,x^{t}\right\}}\text{​}\text{​}p_{s}\left(x^{t\;-\;\tau }\right)\text{​​}\frac{\partial }{\partial w_{ij}}\text{​​}\left(\prod_{u\;=\;1}^{\tau }p\left(x^{t\;-\;u\;+\;1}|x^{t\;-\;u}\right)\gamma^{t}\right)\\ =\underset{\tau \;\to \;\infty }{\lim}\sum_{\left\{x^{t-\tau },x^{t-\tau +1},\cdots ,x^{t}\right\}}\\ p_{s}\left(x^{t\;-\;\tau },x^{t-\;\tau \;+\;1},\cdots ,x^{t}\right)\sum_{u\;=\;1}^{\tau }\frac{\frac{\partial }{\partial w_{ij}}p\left(x^{t\;-\;u\;+\;1}|x^{t\;-\;u}\right)}{p\left(x^{t\;-\;u\;+\;1}|x^{t\;-\;u}\right)}\gamma^{t}\\ \;+\underset{\tau \;\to \;\infty }{\lim}\sum_{\left\{x^{t-\tau },x^{t-\tau +1},\cdots ,x^{t}\right\}}p_{s}\left(x^{t\;-\;\tau },x^{t\;-\;\tau \;+\;1},\cdots ,x^{t}\right)\frac{\partial }{\partial w_{ij}}\gamma^{t}\\ =\underset{\tau \;\to \;\infty }{\lim}\sum_{\left\{x^{t-\tau },x^{t-\tau +1},\cdots ,x^{t}\right\}}\\ p_{s}\left(x^{t\;-\;\tau },x^{t\;-\;\tau \;+\;1},\cdots ,x^{t}\right)\sum_{u\;=\;1}^{\tau }\frac{\frac{\partial }{\partial w_{ij}}p\left(x_{i}^{t\;-\;u\;+\;1}|x^{t\;-\;u}\right)}{p\left(x_{i}^{t\;-\;u\;+\;1}|x^{t\;-\;u}\right)}\gamma^{t}\\ +\sum_{\left\{x^{t-1},x^{t}\right\}}p_{s}\left(x^{t\;-\;1},x^{t}\right)\frac{\partial }{\partial w_{ij}}\gamma^{t}\\ =\underset{\tau \;\to \;\infty }{\lim}{\langle \gamma^{t}\sum_{u\;=\;1}^{\tau }\psi_{i}^{t\;-\;u}x_{j}^{t\;-\;u}\rangle }_{\infty }+{\langle \frac{\partial }{\partial w_{ij}}\gamma^{t}\rangle }_{\infty }. \end{array}$$

We obtain the following derivative with respect to *h*_*i*_ in a similar way:

(14) $$\frac{\partial }{\partial h_{i}}{\langle \gamma^{t}\rangle }_{\infty }=\underset{\tau \to \infty }{\lim}{\langle \gamma^{t}\sum_{u=1}^{\tau }\psi_{i}^{t-u}\rangle }_{\infty }+{\langle \frac{\partial }{\partial h_{i}}\gamma^{t}\rangle }_{\infty }.$$

Calculating the gradients of the objective function, Equation (2), by repeatedly applying the above formula, Equations (13) and (14), we obtain a gradient ascent algorithm $w_{ij}\to w_{ij}+\epsilon \frac{\partial }{\partial w_{ij}}A,\;h_{i}\to h_{i}+\epsilon \frac{\partial }{\partial h_{i}}A$, where

(15) $$\begin{array}{l} \frac{\partial }{\partial w_{ij}}A=\underset{\tau \to \infty }{\lim}{\text{E}}_{p_{s}}[\left({\tilde{\gamma }}_{1}^{t}-{\tilde{\gamma }}_{2}^{t}-{\tilde{\gamma }}_{3}^{t}-{\tilde{\gamma }}_{4}^{t}\right)\\ \;\times \sum_{u=1}^{\tau }\psi_{i}^{t-u}x_{j}^{t-u}-\zeta \left(s_{i}^{t}-s_{0}\right)x_{j}^{t}], \end{array}$$

(16) $$\begin{array}{l} \frac{\partial }{\partial h_{i}}A=\underset{\tau \to \infty }{\lim}{\text{E}}_{p_{s}}[-\left({\tilde{\gamma }}_{1}^{t}-{\tilde{\gamma }}_{2}^{t}-{\tilde{\gamma }}_{3}^{t}-{\tilde{\gamma }}_{4}^{t}\right)\\ \;\times \sum_{u=1}^{\tau }\psi_{i}^{t-u}+\zeta \left(s_{i}^{t}-s_{0}\right)], \end{array}$$

$$\begin{array}{l} {\tilde{\gamma }}_{1}^{t}=\sum_{i}\frac{1}{\text{I}\left[x_{i}^{\nu }|x^{\nu -1}\right]}\log\frac{p\left(x_{i}^{t}|x^{t-1}\right)}{p\left(x_{i}^{t}\right)},\\ {\tilde{\gamma }}_{2}^{t}=\kappa \left\{\frac{1}{2}m^{t}\left(m^{t}-1\right)-\left({\text{E}}_{p_{s}}[m^{\nu }]-{\text{E}}_{p_{s}}\left[x_{i}^{\nu }\right]\right)m^{t}\right\},\\ {\tilde{\gamma }}_{3}^{t}=\eta \sum_{i}\left({\text{E}}_{p_{s}}\left[x_{i}^{\nu }\right]-p_{0}\right)x_{i}^{t},\\ {\tilde{\gamma }}_{4}^{t}=\frac{\zeta }{2}\sum_{i}{\left(s_{i}^{t}-s_{0}\right)}^{2}. \end{array}$$

The last terms in Equations (15) and (16) are due to the explicit dependence of $\tilde{\gamma }$^*t*^_4_ on *w*_*ij*_ and *h*_*i*_, and correspond to the second terms in Equations (13) and (14). Since $\tilde{\gamma }$^*t*^_2_ and $\tilde{\gamma }$^*t*^_3_ do not depend explicitly on *w*_*ij*_ and *h*_*i*_, the corresponding terms are zero. Although $\tilde{\gamma }$^*t*^_1_ depends on *w*_*ij*_ and *h*_*i*_ explicitly, the corresponding terms turn out to vanish as follows:

$$\begin{array}{l} {\langle \frac{\partial }{\partial w_{ij}}\log\frac{p\left(x_{i}^{t}|x^{t-1}\right)}{p\left(x_{i}^{t}\right)}\rangle }_{\infty }\\ \;=\sum_{x^{t},x^{t-1}}p\left(x^{t-1}\right)p\left(x_{\setminus i}^{t}|x^{t-1}\right)\frac{p\left(x_{i}^{t}|x^{t-1}\right)}{p\left(x_{i}^{t}|x^{t-1}\right)}\frac{\partial }{\partial w_{ij}}p\left(x_{i}^{t}|x^{t-1}\right)\\ \;-\sum_{k\in \{0,1\}}\sum_{x_{\setminus i}^{t},x^{t-1}}p\left(x^{t-1}\right)p\left(x_{\setminus i}^{t}|x^{t-1}\right)p\left(x_{i}^{t}=k|x^{t-1}\right)\\ \;\times \frac{\sum_{x_{\setminus i}^{v},x^{v-1}}p\left(x^{v-1}\right)p\left(x_{\setminus i}^{v}|x^{v-1}\right)\frac{\partial }{\partial w_{ij}}p\left(x_{i}^{v}=k|x^{v-1}\right)}{\sum_{x_{\setminus i}^{u},x^{u-1}}p(x^{u-1})p\left(x_{\setminus i}^{u}|x^{u-1}\right)p\left(x_{i}^{u}=k|x^{u-1}\right)}\\ \;=0. \end{array}$$

Here, *x*^*t*^_\*i*_ represents a vector consisting of the components of *x*^*t*^ except the *i*-th component. At this stage, the gradient ascent algorithm, Equations (15) and (16), is not temporarily local while it is composed of synaptically local quantities modulated by biologically plausible global signals. There is, however, a natural approximation by a temporarily local learning rule that exploits the following relation. Notations for exponents and time indices can be clearly discriminated and should not be confounded in the below. For an arbitrary real-valued process *q*^*t*^ and τ > 1, we define 〈 *q*^*t*^〉_τ_ as in the Results section:

(17) $$\begin{array}{l} {\langle q^{t}\rangle }_{\tau }=\frac{1}{\tau }q^{t-1}+\left(1-\frac{1}{\tau }\right){\langle q^{t-1}\rangle }_{\tau },\text{ and}\\ {\langle q^{t}\rangle }_{\tau }=0\;(t\leq 0). \end{array}$$

Then

$${\langle q^{t}\rangle }_{\tau }=\frac{1}{\tau }\sum_{u=0}^{\infty }{\left(1-\frac{1}{\tau }\right)}^{u}q^{t-u-1}.$$

Here, we put *q*^*t*^ = 0 for *t* < 0. If the process under consideration is stationary, 〈 *q*^*t*^〉_τ_ approaches the long-time average of *q*^*t*^ as τ → ∞ and *t*/τ → ∞. Similarly, assuming that γ^*t*^_*k*_ has little correlation with ψ^*t* − τ^_*i*_ for sufficiently large τ ≫ 1, and that *T* ≫ τ, we have

(18) $$\begin{array}{l} \underset{\tau \to \infty }{\lim}{\langle \gamma^{t}\sum_{u=1}^{\tau }\psi_{i}^{t-u}x_{j}^{t-u}\rangle }_{\infty }\approx {\langle \tau \gamma^{t}{\langle \psi_{i}^{t}x_{j}^{t}\rangle }_{\tau }\rangle }_{T}\\ \;\approx \frac{\tau }{T}\sum_{t=1}^{T}\gamma^{t}{\langle \psi_{i}^{t}x_{j}^{t}\rangle }_{\tau }. \end{array}$$

Exploiting these relations, Equations (17) and (18), instead of explicitly handling time averages, we obtain an approximation of the gradients in Equations (15) and (16) which is calculated in a temporarily local manner. Then, applying the usual stochastic approximation theory (Robbins and Monro, 1951), we obtain the temporarily local learning rule, Equations (3) and (4). Since our goal is to study biologically plausible learning rules and not to obtain rigorous convergence, we have not adjusted the value of ϵ depending on *t* in the simulations. For a fixed small ϵ, however, the learning has essentially stopped, probably at a local maximum.

### 4.3. Autocorrelograms and coefficients of variation

We calculated autocorrelograms and CVs of ISIs in Figures 2J,K. For this calculation, we used firing activity for a particular *T*_0_ = 50000 steps (*T*_1_ ≤ *t* ≤ *T*_1_ + *T*_0_) after learning. From this spike train, the autocorrelogram *C*(ρ) with lag ρ for the *i*-th neuron is calculated as

$$C(\rho )=\frac{1}{T_{0}-\rho }\sum_{t=T_{1}+\rho }^{T_{1}+T_{0}}x_{i}^{t}x_{i}^{t-\rho }.$$

The CVs of ISIs of the *i*-th neuron is calculated from the collection of ISIs {*l*_*u*_}_1 ≤ *u* ≤ *U*_ in the same *T*_0_ steps as

$$SD(l_{u})/mean(l_{u}),$$

where

$$mean\left(l_{u}\right)=\frac{1}{U}\underset{u}{\sum }l_{u},\;\;\;SD\left(l_{u}\right)=\sqrt{\frac{1}{U}{\left(\underset{u}{\sum }l_{u}-mean\left(l_{u}\right)\right)}^{2}}.$$

### 4.4. Image preparation in the learning of natural images

The original and preprocessed images are the same as those used in the seminal paper (Olshausen and Field, 1997). The original images are ten 512× 512 images of natural surroundings in the American northwest. Gray-scaled images are zero-centered, whitened and low-pass-filtered using a filter with the frequency response

$$L(f)=f\exp\left(-{\left(f/f_{c}\right)}^{4}\right),$$

where the cut-off frequency is *f*_*c*_ = 200 cycles per picture. Whitening and zero-centering are due to the high-pass filtering property of *f*, while exp (−(*f* / *f*_*c*_)^4^) eliminates artifacts of higher frequency than rectangular sampling (see Atick, 1992 for further details). The images and the Matlab program codes are provided at the webpage by Olshausen (2012).

### 4.5. An approximate measure of the mutual information

Following the preceding study (Tanaka et al., 2009), we define an approximate measure of the mutual information $I_{gauss}=\log|C|-\frac{1}{2}\log|D|$. *C* and *D* are covariance matrices of *x*^*t*^ and *x*^*t*^ ⊗ *x*^*t* − 1^, respectively. Particularly, *C* is a *N* × *N* matrix defined as $C_{ij}={\langle x_{i}^{t}x_{j}^{t}\rangle }_{\infty }-{\langle x_{i}^{t}\rangle }_{\infty }{\langle x_{j}^{t}\rangle }_{\infty }$ for 1 ≤ *i*, *j* ≤ *N*, while *D* is a 2*N* × 2*N* matrix defined as $D_{ij}={\langle x_{i}^{t}x_{j}^{t}\rangle }_{\infty }-{\langle x_{i}^{t}\rangle }_{\infty }{\langle x_{j}^{t}\rangle }_{\infty },D_{i,j+N}={\langle x_{i}^{t}x_{j}^{t-1}\rangle }_{\infty }-{\langle x_{i}^{t}\rangle }_{\infty }{\langle x_{j}^{t-1}\rangle }_{\infty }$, $D_{i+N,j}={\langle x_{i}^{t-1}x_{j}^{t}\rangle }_{\infty }-{\langle x_{i}^{t-1}\rangle }_{\infty }{\langle x_{j}^{t}\rangle }_{\infty }$ and $D_{i+N,j+N}={\langle x_{i}^{t-1}x_{j}^{t-1}\rangle }_{\infty }-{\langle x_{i}^{t-1}\rangle }_{\infty }{\langle x_{j}^{t-1}\rangle }_{\infty }$ for 1 ≤ *i*, *j* ≤ *N*. If both of the firing distribution of *x*^*t*^ and the joint distribution of *x*^*t*^ and *x*^*t* − 1^ have Gaussian probability densities, the mutual information I [*x*^*t*^; *x*^*t* − 1^] is equal to *I*_*gauss*_. In the numerical simulation, we have calculated a finite-time approximation $\hat{I}$_*gauss*_ by replacing the terms like 〈 *x*^*t*^_*i*_*x*^*t*^_*j*_〉_∞_ with corresponding finite-time averages like ${\langle x_{i}^{t}x_{j}^{t}\rangle }_{T}$.

### 4.6. Calculation of spike triggered averages of input images

In the learning of input images, we calculated reverse correlations by taking spike-triggered average of input images presented at the previous time step of neuronal firing, as in the examination of receptive fields in physiological experiments (Ringach and Shapley, 2004). Concretely, the spike-triggered average of input images with respect to neuron *i* is calculated as *y*^(*i*)^_STA, *j*_ = 〈 *y*^*t* − 1^_*j*_ *x*^*t*^_*i*_〉_*T*_ for 1 ≤ *i* ≤ *N* and 1 ≤ *j* ≤ 144. Then, we linearly rescale *y*^(*i*)^_STA, *j*_ into y^*, (*i*)^_STA, *j*_ for each *i* so that the mean over the pixel components is 128 as $y_{\text{mean}}^{*,(i)}=\frac{1}{144}\sum_{j}y_{\text{STA},j}^{*,(i)}=128$, and that the maximal deviation from the mean is 127 as max_*j*_|y^*, (*i*)^_STA, *j*_ − y^*,(*i*)^_mean_| = 127. We have plotted the rescaled average y^*, (*i*)^_STA, *j*_ as a 12× 12 pixel patch in a gray scale ranging from 1 (black) through 255 (white) in Figure 5B.

### 4.7. Interpretation of the internal variable as an input current

We have defined our dynamics as $p\left(x_{i}^{t+1}=1|s_{i}^{t}\right)=p_{\text{max}}/\left\{1+\exp\left(-s_{i}^{t}\right)\right\}$. The internal variable *s*^*t*^_*i*_ can be interpreted as an input current to the neuron *i*. To show this, we simulate an integrate-and-fire neuron model with constant input currents. We solve the following continuous-time dynamics of membrane potential *V* by the Euler-Maruyama method with a step size Δ *t* = 0.01 (ms):

$$\begin{array}{l} \tau_{m}\text{d}V=(-V+V_{\text{rest}}+I)\text{d}t+D\text{d}\xi .\\ \;V\leftarrow V_{\text{rest}}\text{ if }V>\theta \end{array}$$

**Figure 8:**
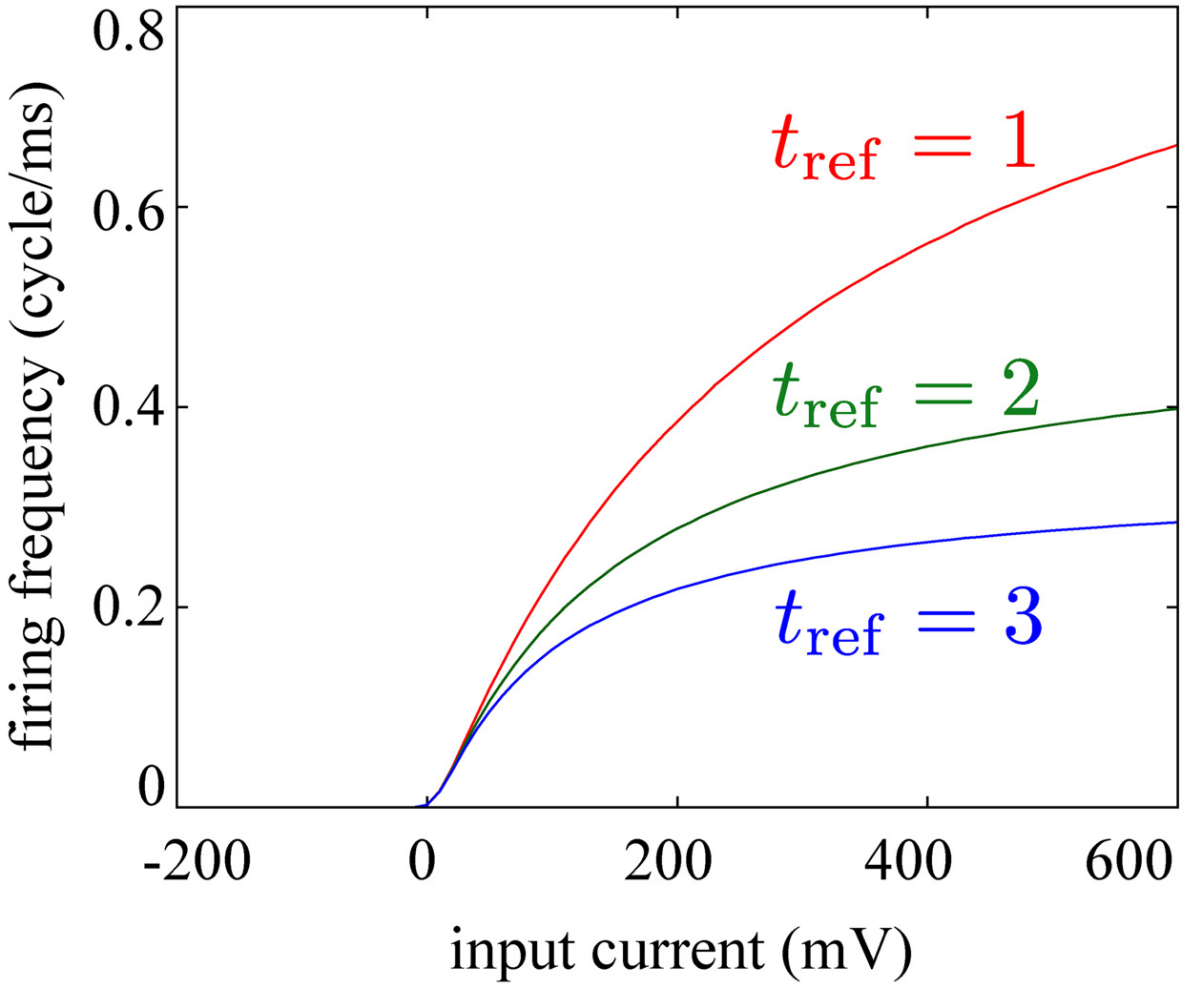
**Dependence of firing frequencies of an integrate-and-fire model on magnitudes of constant input currents falls on a sigmoid curve**. Saturating frequencies depend on the length of the refractory period *t*_ref_ as indicated by the differently-colored curves [red: *t*_ref_ = 1 (ms), green: *t*_ref_ = 2 (ms), blue: *t*_ref_ = 3 (ms)].

In the above equation, we have added Wiener noise ξ with a constant coefficient *D* = 3. The membrane constant is τ_*m*_ = 15 (ms). If *V* exceeds the threshold potential θ = −50 (mV), we reset the membrane potential to the resting potential *V*_rest_ = −70 (mV) and hold it for a refractory period *t*_ref_ = 1, 2, or 3 (ms). We apply input currents *I* = −200~ 600 (mV) to this model, describing the input current in units of mV on the assumption that the input resistance is a dimensionless quantity equal to unity. Then, we find that the dependence of the firing frequency *f* (cycle/ms) of the neuron model on the applied current *I* falls on a sigmoid curve as shown in Figure 8. We further observe that the maximal firing probability depends on the length of the refractory period (Figure 8).

## Funding

This study was supported by Grants-in-Aid from The Ministry of Education, Culture, Sports, Science and Technology (MEXT) for Scientific Research (22300113 and 25250006 to Takeshi Kaneko); for Exploratory Research (23650175 to Takeshi Kaneko); and for Scientific Researches on Innovative Areas “Mesoscopic Neurocircuitry” (23115101 to Takeshi Kaneko and 25115719 to Toshio Aoyagi) and “The study on the neural dynamics for understanding communication in terms of complex hetero systems” (21120002 to Toshio Aoyagi).

### Conflict of interest statement

The authors declare that the research was conducted in the absence of any commercial or financial relationships that could be construed as a potential conflict of interest.
